# *In vitro* selections of mammaglobin A and mammaglobin B aptamers for the recognition of circulating breast tumor cells

**DOI:** 10.1038/s41598-017-13751-z

**Published:** 2017-11-03

**Authors:** Eman M. Hassan, William G. Willmore, Bruce C. McKay, Maria C. DeRosa

**Affiliations:** 1 Institut national de la recherche scientifique - Energie, Materiaux Telecommunication 1650 boul. Lionel-Boulet, Varennes, Quebec, J3X1S2 Canada; 2 Institute of Biochemistry, Carleton University, 1125 Colonel By Drive, Ottawa, Ontario, K1S 5B6 Canada; 3 Department of Biology, Carleton University, 1125 Colonel By Drive, Ottawa, Ontario, K1S 5B6 Canada; 4 Department of Chemistry, Carleton University, 1125 Colonel By Drive, Ottawa, Ontario, K1S 5B6 Canada

## Abstract

Mammaglobin B (MGB2) and mammaglobin A (MGB1) are proteins expressed in metastatic breast cancers. The early detection of circulating tumor cells (CTCs) in breast cancer patients is crucial to decrease mortality rate. Herein, novel aptamers were successfully selected and characterized against MGB2 and MGB1 proteins using a hybrid SELEX approach. The potential use of the selected aptamers in breast CTC detection was studied using spiked breast cancer cells in whole blood lysate. The results obtained from this study showed that the selected aptamers (MAMB1 and MAMA2) bind to their target breast cancer cell lines with high affinity (low nanomolar K_d_ values) and specificity. They also bind to their free recombinant target proteins and show minimal non-specific binding to normal and other cancer cell lines. Additionally, they were able to distinguish a low number of breast cancer cells spiked in whole blood lysate containing normal blood cells. The results obtained in this study indicate the great potential for the use of aptamers to detect MGB1 and MGB2 protein biomarkers, expressed on the surface of breast CTCs.

## Introduction

Cancer is a complex disease that originates as a result of multiple genomic mutations leading to a disruption of normal cellular homeostasis^1^. Breast cancer is the most common cancer diagnosed in women, with an estimated 1.67 million new cases diagnosed worldwide in 2012^2^. One in eight U.S. women (about 12%) will develop invasive breast cancer over the course of their lifetime^3^. In 2016, an estimated 246,660 new cases of invasive breast cancer are expected to be diagnosed in women in the U.S^3^. Metastasis is the main cause of death for the majority of breast cancer patients^4^. To date, few biomarkers are used to detect metastatic breast cancer^5–7^. Further identification of promising biomarkers would be of great benefit in the field of breast cancer diagnosis and therapy.

Circulating tumor cells (CTCs) are cells that are shed from the primary tumor and start to invade surrounding tissues, intravasate into the blood stream to circulate with its components, extravasate to distant tissues in different organs, start to adapt to the new microenvironment, and proliferate starting a secondary tumor^8^. To initiate metastasis, cancer cells transform from an epithelial form to a mesenchymal form in a process known as epithelial-to-mesenchymal transition (EMT)^9^. In EMT, cancer cells gain new properties including increased invasive capacity, higher resistance to apoptosis, and a noticeable increase in the extracellular matrix (ECM) components^10,11^. The changes are reversible and, once the cancer cells reach their destination, they regain their epithelial properties in a process called mesenchymal-to-epithelial transition (MET)^12^.

Mammaglobin B (MGB2) and Mammaglobin A (MGB1) are secretory proteins and members of the uteroglobin gene family^13,14^. Both are small proteins (approximately 10 kDa) that contain an alpha-helix in their structure and are often found as dimers^15,16^. Little is known about their function, but it is believed that they have a role in cancer development, immune system regulation, and the transport of aromatic molecules, such as steroid hormones^17^. MGB2 and MGB1 have been reported to be highly homologous (58% homology) and are thought to perform the same biological functions^13^. MGB2 is mostly expressed in mucosal tissues and is found at high levels in many secretions including those from uterine, prostatic, pulmonary, and lacrimal and salivary glands^13,14,18,19^. MGB2 is overexpressed in ovarian and endometrium cancers, as well as all primary and metastatic breast cancers^20–22^. In contrast, MGB1 overexpression is only limited to breast cancer^22,23^. Both of the proteins have been reported as markers of breast cancer micro-metastases to lymph nodes and markers of CTCs found in the blood of breast cancer patients^24–29^.

The development of highly specific recognition probes against MGB2 and MGB1 will aid in the diagnosis of breast cancer and CTCs from breast cancer tumors. Aptamers are powerful molecular recognition probes^30^. They are synthetic, short (∼15–100 nucleotides in length) single stranded DNA or RNA oligonucleotides that recognize molecular targets, such as biomarkers, through a unique three-dimensional interaction with the target with high affinity and specificity^30^. Aptamers are produced via an *in vitro* selection method called Systematic Evolution of Ligands by EXponential enrichment (SELEX) by the repetitive partitioning of binders from a large library of oligonucleotides having an initial diversity of 10^13^–10^15^ random sequences^30,31^. Each round of aptamer selection in SELEX is performed by binding and eluting aptamers from target molecules or cells, leading to the selection of aptamers from the library with high affinity and specificity for their targets^32,33^. Compared to their broadly-used antibody counterparts, aptamers possess unique properties in that they can be easily synthesized and have the ability to be chemically modified, which makes aptamers more convenient to use as molecular probes for various applications^34–36^.

Cell-SELEX is a powerful tool for the identification of new biomarkers^33^. However, the cell has a large number of surface molecules and potential target biomarkers, making potential nonspecific interactions of great concern^37,38^. Many additional steps have been introduced to cell-SELEX to overcome this obstacle, including counter selection steps^39^. Hybrid, or cross-over cell-SELEX, allows for the selection of specific biomarkers on the surface of the cell by alternating the SELEX process between a purified recombinant protein of interest, and the cells that express this protein, as targets^40,41^. Unlike whole cell-SELEX, where the knowledge of the biomarker is not necessary to develop aptamers specific for a certain cell line, hybrid SELEX determines the biomarker of interest before the SELEX process takes place^42^. Hybrid SELEX produces highly enriched aptamers against the target of interest (biomarker) on the surface of the cell. Moreover, aptamers selected by hybrid SELEX are potentially more specific to the tumor cell line, versus the control, than aptamers developed utilizing whole cells as targets^42^.

**Figure 1:**
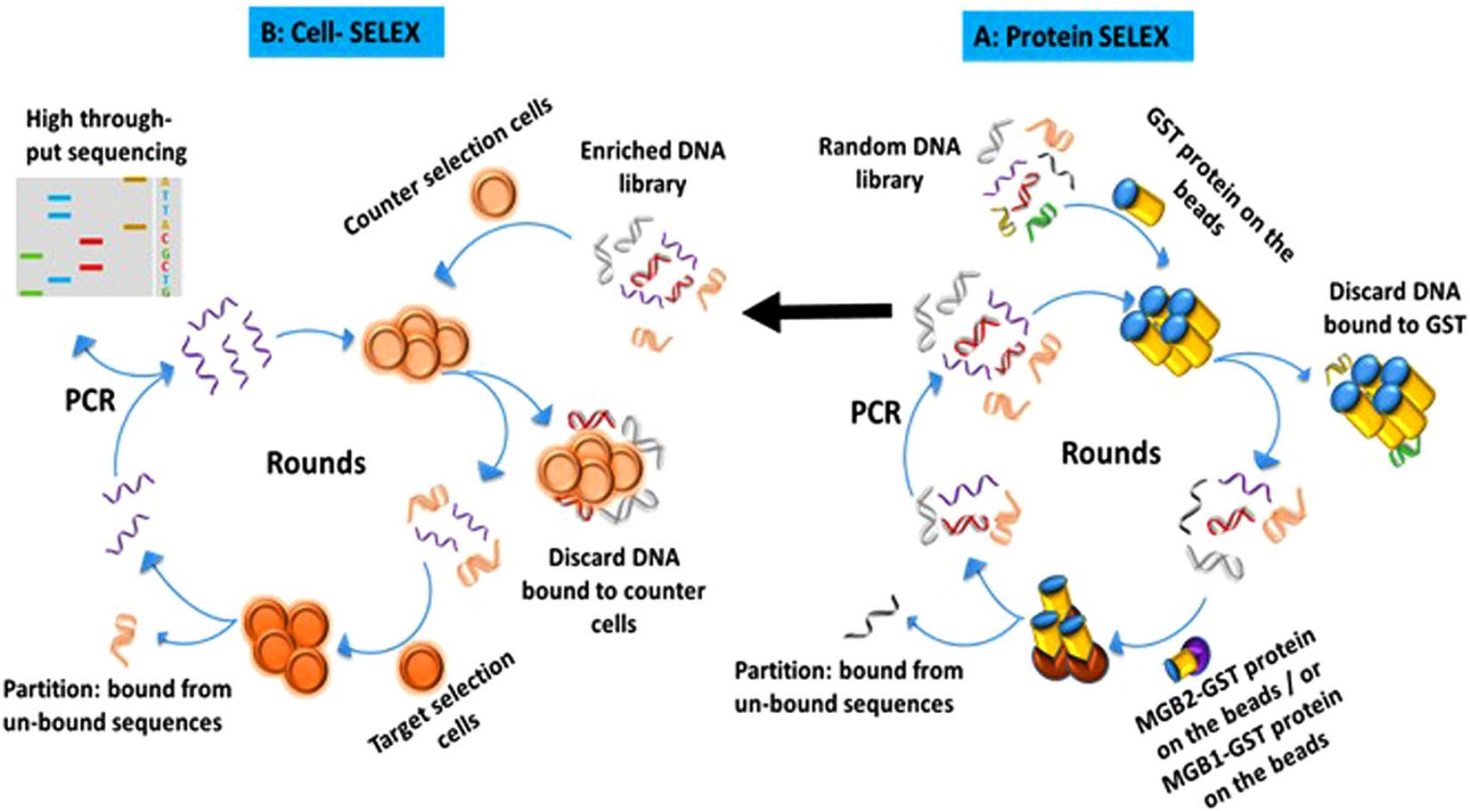
Schematic diagram of hybrid SELEX method for selection of MGB2 and MGB1-specific DNA aptamers. Hybrid SELEX is divided into **(A)** a protein-based SELEX in which GST-MGB2 conjugated beads (brown) and GST-MGB1 proteins conjugated beads (purple) were used. The protein SELEX was done for 21 rounds for MGB2 and 4 round for MGB1. Subsequently, the enriched pools of both proteins were transferred to cells **(B)** to start cell-based SELEX in which HCAEC and MCF10A were used as counter selection cell lines. MCF7 was used as the target selection cell line for MGB2 and MDA-MB-415 was used as the target selection cell line for MGB1. After 7 rounds of cell-SELEX to each target cell line, the final pools for both targets were transferred back to the proteins for another 2 additional rounds for each MGB2 and MGB1 to make the library more specific to MGB2 and MGB1.

In this study, a series of aptamers against MGB2 and MGB1 was developed, for the first time, using the hybrid cell-SELEX approach (Fig. 1). Characterization of the selected aptamers, against relevant cell targets and the free recombinant protein, were evaluated using flow cytometry, fluorescence microscopy, and electrophoretic mobility shift assay. Selected aptamers were used to test their ability to detect breast cancer cells in plasma and whole blood lysate-like environments. The ability of the aptamers to detect spiked breast cancer cells in a mixture of whole blood lysate containing normal blood cells was investigated. Additionally, cell-aptamer binding studies were performed to verify the protein target of the aptamers selected.

## Results and Discussion

### Hybrid SELEX analysis: Development of aptamers that bind specifically to MGB2 and MGB1 proteins

#### Monitoring the enrichment of the ssDNA library during protein- and cell-SELEX

**Figure 2:**
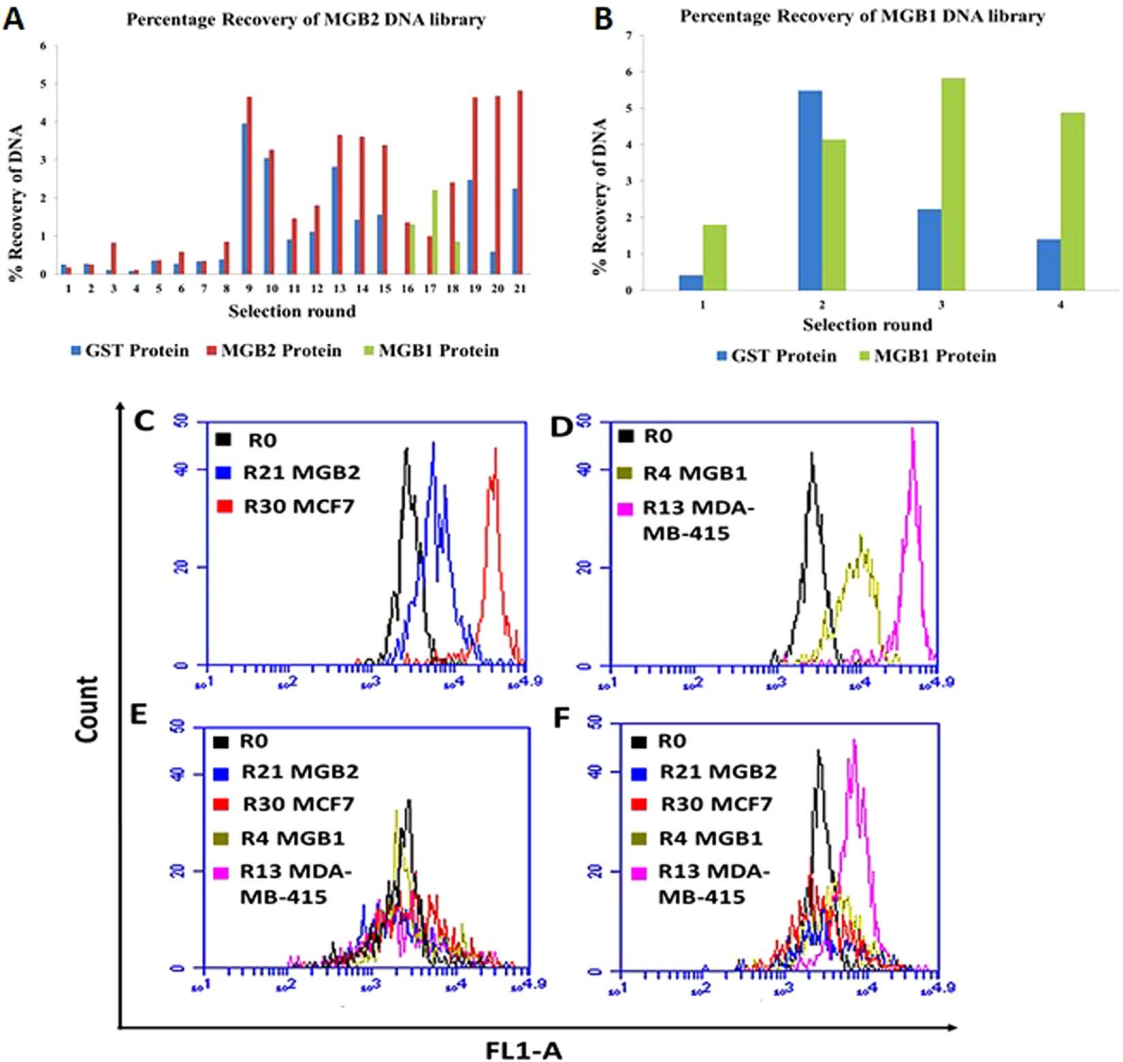
Monitoring the enrichment of ssDNA library of MGB2 and MGB1 during hybrid SELEX. (**A**) Percentage recovery of MGB2 library during protein SELEX. GST-protein (blue) was used for counter selection throughout the selection. MGB1 protein (green) was used as the counter selection target in R16 to R18 to split the main library into two parts to eliminate the sequences that could bind to MGB1 and MGB2 targets together. (**B**) Percentage recovery of MGB1 protein library during protein SELEX. R17 counter selection (green) from MGB2 SELEX was used as the initial ssDNA library of MGB1 SELEX. The percentage recovery for both proteins SELEX was measured as the emission of FAM labelled ssDNA libraries at 520 nm. (**C**) The fluorescence intensity (FL1-A) of MCF7 (MGB2 positive cell line) when binding to R0, R21, and R30 of MGB2. (**D**) The fluorescence intensity (FL1-A) of MDA-MB-415 (MGB1 positive cell line) when binding to R0, R4, and R13 of MGB1. (**E**,**F**) The fluorescence intensity (FL1-A) of HCAEC cells and MCF10A (counter cell lines) respectively when binding to R0, R21 MGB2, R4 MGB1, R30 MCF7 and R13 MDA-MB-415. The fluorescence intensity was measured using flow cytometry by incubating the 6-FAM tagged ssDNA with the cells for 30 min at 4 °C in PBS. The fluorescence intensity of cells from all cell lines (control/background signal) was subtracted from the fluorescence intensity of the positive and counter cell lines and included in the graph.

In this study, we modified the cell-SELEX protocol^37^ by introducing a purified protein SELEX before initiating cell-SELEX (hybrid SELEX) to enrich the ssDNA library for aptamers with specificity towards MGB2 and MGB1. The percentage recovery of the ssDNA libraries (during protein SELEX) for both recombinant glutathione *S*-transferase (GST)-tagged MGB2 and MGB1 was monitored by measuring fluorescein emission (520 nm) from aptamer fractions eluted from glutathione modified beads (Fig. 2A and B). Figure 2A shows an increase in the percentage recovery of DNA with increasing numbers of selection rounds of MGB2, while recovery of DNA from GST-protein decreased from an initial high amount to remain consistently lower than the positive elutions. No increase in recovery from positive elutions was seen after rounds 13 to 15, suggesting that enrichment reached a maximum at round 13. Due to the fact that MGB2 and MGB1 are similar, MGB1 was used as a counter selection to eliminate cross-reacting sequences (rounds 16, 17 and 18) resulting in the division of the ssDNA library in almost two equal parts (splitting the main ssDNA library), as indicated by the percentage recovery for MGB2 (Round 16) shown in Fig. 2A. This is matched our expectations, since MGB2 and MGB1 are 58% alike^13^. The Round 17 counter selection pool was used as the initial ssDNA library for MGB1 SELEX due to its higher enrichment with MGB1-binding sequences (Fig. 2A and B). The final DNA percentage recovery eluted from both proteins was similar, indicating high enrichment (around 4.8%).

To develop aptamers against metastatic breast cancer, MCF7 and MDA-MB-415 breast cancer cell lines derived from metastatic sites were used in the cell-SELEX and the expression of MGB2 and MGB1 in both cell lines, as well as the counter selection cell lines (MCF10A and HCAEC), was evaluated using ELISA. Our results confirmed that the MGB2 and MGB1 proteins are exclusively expressed on the surface of MCF7 and MDA-MB-415 cells respectively, compared to other cell lines used in the SELEX (Supplementary Fig. S1) which confirms the results found in previous studies^13,14^.

Flow cytometry was used to monitor the enrichment of ssDNA libraries during cell-SELEX. A clear shift in fluorescence intensity was observed in Round 30 of MCF7 (MGB2) compared to Round 21 (end of protein-SELEX) and Round 0 (Fig. 2C). Higher fluorescence intensity shift was observed in R13 of MDA-MB-415 (MGB1) compared to R4 (end of protein-SELEX) and R0 (Fig. 2D). The counter selection cell lines did not show a change in the fluorescence intensity, indicating the specificity of the aptamers selected for the MCF7 and MDA-MB-415 cell lines (Fig. 2E and F).

### High throughput sequencing (HTS) and bioinformatics analysis

Our SELEX method included a number of targets (proteins and cell lines) and therefore HTS was preferable due to its many advantages over traditional cloning and sequencing^43^. Specifically, the ability to efficiently compare sequences from multiple rounds with varying conditions in a statistically robust fashion. The high throughput datasets for both targets were grouped into clusters using AptaCluster software, according to their similarity. Each cluster contained sequences that were arranged according to their enrichment and count. The overall analysis of MGB2 and MGB1 sequences showed that MBG2 aptamers seemed to be more C-rich, while MGB1 aptamers were more T-rich, compared to aptamers in Round 0 (according to bioinformatics analysis using AptaCluster as a software). The analysis of the random region (N) of both MGB2 and MGB1 sequences showed that the dominant length of N was 25 bases (one more than what was present in the original synthesized pool) and 24 bases for MGB2 and MGB1 sequences respectively, indicating that mutations took place during the selection process, for example during PCR amplification (Supplementary Fig. S2). Comparing enrichment of a sequence (rather than just the copy number) from early to later selection rounds should minimize artifacts from PCR bias and allow for the choice of sequences with improved aptamer-target binding. Twelve MGB2 and eight MGB1 aptamers were chosen from the final pools for further study, based on their enrichment in the last round of selection, compared to Round 0. The secondary structure of all candidate aptamers was predicted using RNA structure software (Supplementary Fig. S3).

### Screening for the binding affinity of the selected aptamers and K_d_ determination

**Figure 3:**
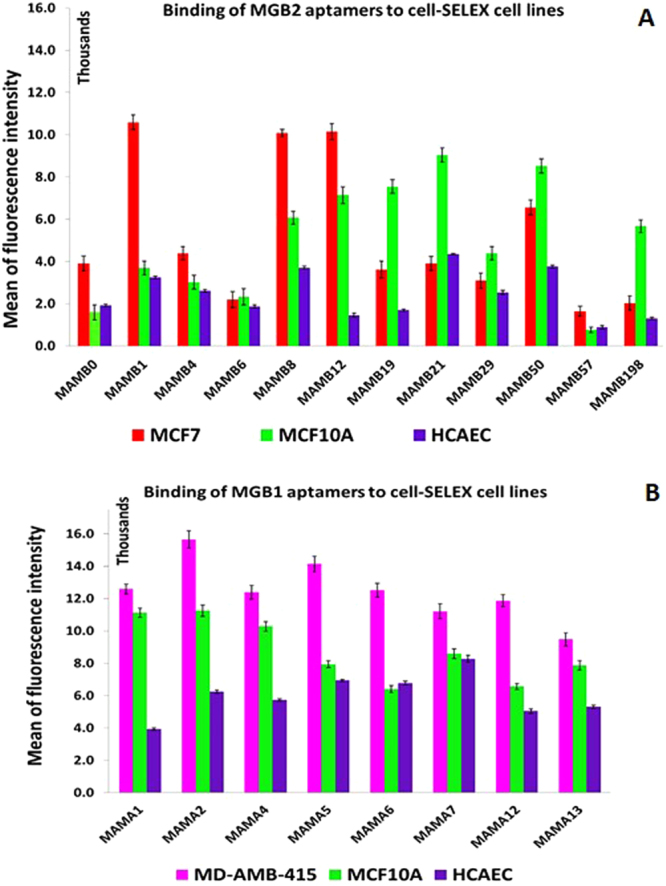
Screening results of the aptamer candidates against cell-SELEX cell lines using flow cytometry. (**A**) Screening results of MGB2 aptamers show six aptamers out of twelve (MAMB0, MAMB1, MAMB4, MAMB8, MAMB12, MAMB57) with higher fluorescence intensity in the target selection cell line than counter selection ones. (**B**) Screening results of MGB1 aptamers show four aptamers out of eight (MAMA2, MAMA5, MAMA6, and MAMA12) with higher fluorescence intensity in the target selection cell line than counter selection ones. Promising aptamers were chosen for further studies. The experiments were repeated twice. Values are shown as means ± S.E.M.

**Figure 4:**
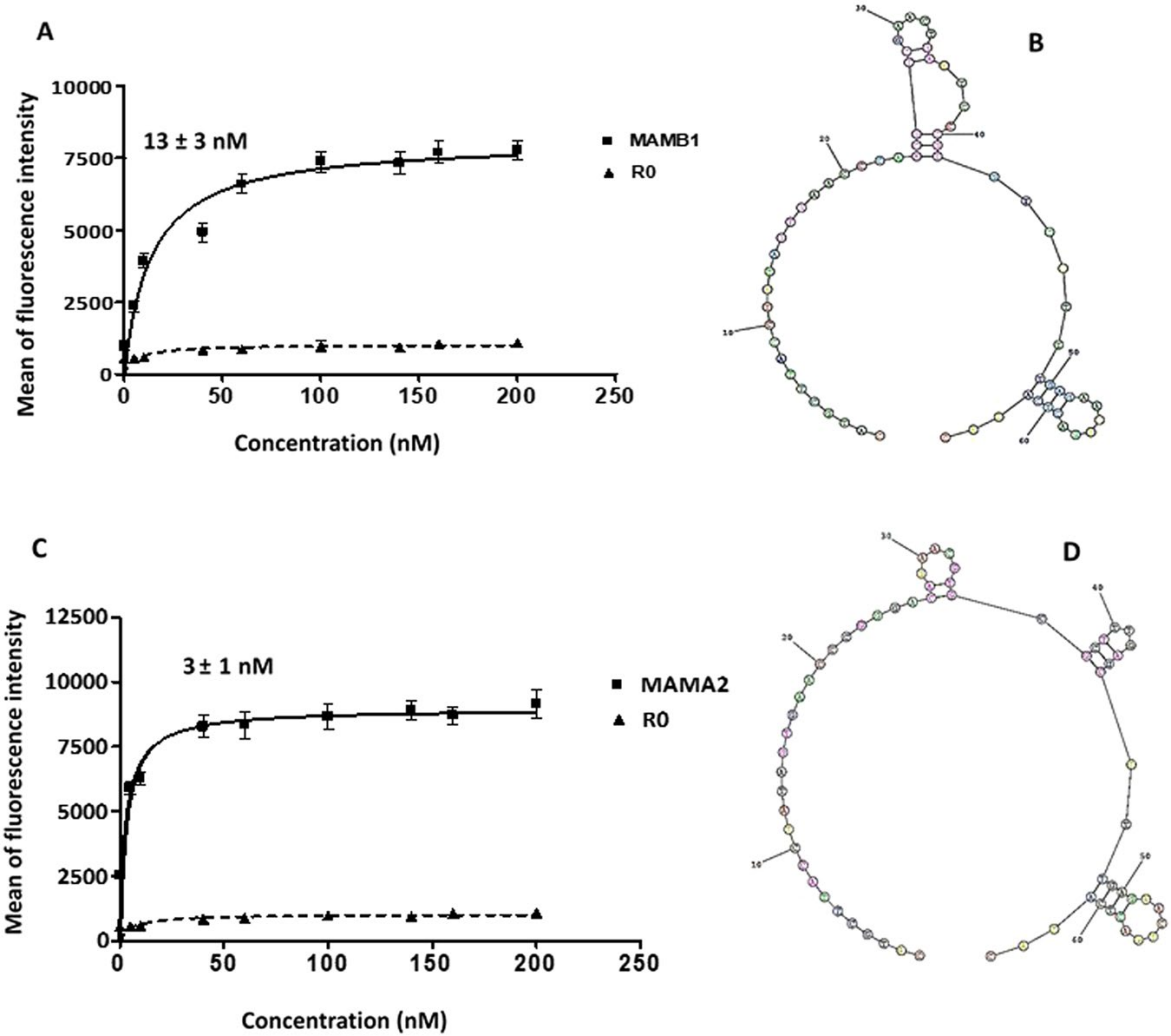
Binding curves of 6-FAM-labeled aptamers to MCF7 and MDA-MB-415 target cell lines. (**A**) MAMB1 K_d_ curve: MCF7 cells were incubated with increasing concentrations (nM) of MAMB1 then was evaluated by flow cytometry. (**B**) The predicted secondary structure of MAMB1 aptamer using RNAstructure software. (**C**) MAMA2 K_d_ curve: MDA-MB-415 cells were incubated with increasing concentrations (nM) of MAMA2 then was evaluated by flow cytometry. (**D**) The predicted secondary structure of MAMA2 aptamer using RNAstructure software. To calculate the apparent K_d_ of the interaction of MAMB1 and MAMA2 to their corresponding cell lines, the mean fluorescence intensity of the aptamer-cell dissociation vs. concentration was fit to the equation Y=Bmax X/(K_d_+X). R0 ssDNA library was used as control. The Kd graphs are the average of three trials. Values are shown as means ± S.E.M.

Applying the hybrid SELEX methodology allowed us to obtain aptamers that were expected to be specific both to the target protein and to tumor cells expressing the same target biomarker (in this case, MGB2 or MGB1). To evaluate the binding affinity, all selected aptamers were labelled with 6-FAM on the 5′ end and were subjected to flow cytometry analysis against the target and counter selection cell lines. As Fig. 3 shows, six out of twelve aptamers of MGB2 and four out of eight of MGB1 aptamers showed higher binding affinity to MCF7, compared to MCF10A and HCAEC cell lines (Fig. 3A and B). These aptamers were selected for further characterization to determine their K_d_ values (against their target cell lines) (Table 1 and Supplementary Table 1). All K_d_ values obtained here were in the nanomolar range, which is typical for aptamers developed against cancer cells using cell-SELEX^44^. MGB2 selected aptamers showed K_d_ values in the low nanomolar range (from 13 to 105 nM; Table 1). MAMB1 and MAMB12 showed the highest affinity (lowest K_d_ values) (Table 1) of 14 ± 3 nM and 20 ± 6 nM respectively (Fig. 4A). However, all MGB1 selected aptamers showed nanomolar affinity for the MDA-MB-415 cell line, with K_d_ values from 3 to 54 nM (Table 1). MAMA12 and MAMA2 aptamers showed the lowest K_d_ values, with values of 8 ± 3 nM and 3 ± 1 nM for MAMA12 and MAMA2, respectively. This indicated that both aptamers have a high affinity for the MDA-MB-415 cell line (Fig. 4C). Notably, their predicted secondary structures were very simple (Fig. 4B and D), however, the structure of these aptamers may become more complex when interacting with their targets.

**Table 1:** Aptamer sequences of MGB2 and MGB1 targets and their corresponding K_d_ values.

Aptamer	Aptamer sequence (5′-3′)^a^	K_d_ (nM)
MAMB0	CATGCTTACCTATAGTGAACCCCAACACACGTGTAGATCCTGCGGCTTTGAGAACTGACTCATAC	80 ± 14
MAMB1	CATGCTTACCTATAGTGAACCCAACGTCGAACTGAATCCCGTGTCCTTTGAGAACTGACTCATAC	13 ± 3
MAMB4	CATGCTTACCTATAGTGAACGCGGCATGTTGGCATCTTGGTCCTGCTTTGAGAACTGACTCATAC	105 ± 20
MAMB8	CATGCTTACCTATAGTGAACCACGACACGCGCGATCGTCTCACTGCTTTGAGAACTGACTCATAC	78 ± 15
MAMB12	CATGCTTACCTATAGTGAACCCACCACACAGCGGATACACCATGGCTTTGAGAACTGACTCATAC	19 ± 5
MAMB57	CATGCTTACCTATAGTGAACCCGAAGAGGATGTGCGGTCCCATTGCTTTGAGAACTGACTCATAC	72 ± 9
MAMA2	CATGCTTACCTATAGTGAACCCGGGACAGAACGTGCGCTTTGAGCTTTGAGAACTGACTCATAC	3 ± 1
MAMA5	CATGCTTACCTATAGTGAACGGTTGGCATCTTGGTCCTGCTTTGCTTTGAGAACTGACTCATAC	54 ± 13
MAMA6	CATGCTTACCTATAGTGAACTGTTGGCATCTTGGTCCTGCTTTGCTTTGAGAACTGACTCATAC	49 ± 20
MAMA12	CATGCTTACCTATAGTGAACGTTGGCATCTTGGTCCTGCTTTGACTTTGAGAACTGACTCATAC	8 ± 3

### Binding selectivity of MAMB1, MAMB12 and MAMA2, MAMA12 aptamers to cancer and normal cell lines

The binding selectivity was further tested for MAMB1, MAMB12, and MAMA2, MAMA12 aptamers for normal and cancer cell lines. As expected, MAMB1 and MAMB12 bound with highest affinity to their target cell line MCF7, but no binding was observed to the other breast cancer cell lines MDA-MB-415 and the triple negative MDA-MB-213, which are known to not express MGB2 protein^13,14^. MGB2 is expressed in different tissues^13,17,18^ and therefore MGB2 selected aptamers (MAMAB1 and MAMB12) showed low level of binding to cell lines other than breast cancer cell lines, such as HeLa and HepG2 cells (Table 2). MGB1 aptamers (MAMA2 and MAMA12) were found to only recognize breast cancer cells due to the fact that the MGB1 protein is only expressed in breast tissue; making it a potentially specific biomarker.

**Table 2:** Selectivity of MAMB1, MAMB12, MAMA2, MAMA12 aptamer to other normal and cancer cell lines.

*Cell line*	*MAMB1*	*MAMB12*	*MAMA2*	*MAMA12*
***MCF7***	++++	++++	+	+
***WI38***	+	+	−	−
***HEK-293***	+	+	−	−
***MDA-MB-415***	−	−	+++	+++
***HeLa***	+	−	−	−
***HepG2***	+	+	−	−
***MDA-MB-231***	−	−	−	−

The selectivity of the chosen aptamers to the different cell lines were calculated based on the relative binding capacity of the target cell line. A cut off 15% was determined according to the binding capacity. −<15, + >15–30%, ++ >30–45%, +++>45–60%, ++++>60–75%.

### Binding of MAMB1 and MAMA2 to their target breast cancer cells by fluorescence microscopy

**Figure 5:**
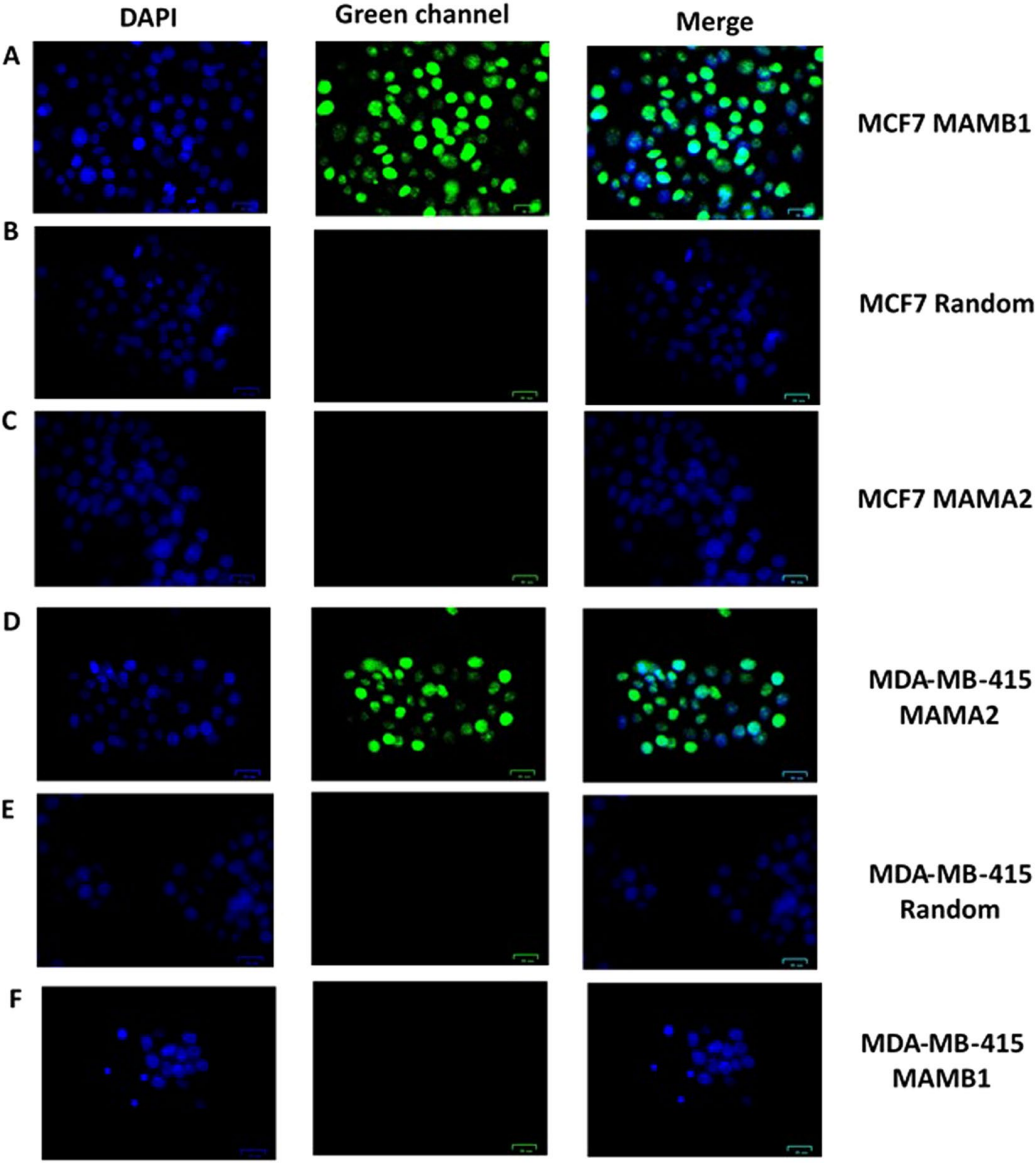
Fluorescence microscopy images of aptamers MAMB1 and MAMA2 binding to target breast cancer cell lines. (**A**) MAMB1 binding to MCF7 cells, (**B**) random sequence binding to MCF7, (**C**) MAMA2 binding to MCF7. (**D**) MAMA2 binding to MDA-MB-415 cells, (**E**) random sequence binding to MDA-MB-415, (**F**) MAMB1 binding to MDA-MB-415. Both aptamers and the random sequence were incubated with the cells for 30 min and then imaged. Scale bars correspond to 25 μm in all images.

MAMB1 and MAMA2 aptamers showed high specific binding to their target breast cancer cells lines MCF7 and MDA-MB-415 respectively (Fig. 5A and D). MAMB1 did not show any binding to MDA-MB-415 (Fig. 5F), and same was observed with MAMA2 against MCF7 (Fig. 5C), which matches our expectations since the pool was divided into two parts when MGB1 was used as a counter selection target in the protein-SELEX. No binding was observed when both aptamers were incubated with a random sequence (dopamine aptamer) (Fig. 5B and E). No affinity of both aptamers was observed when they were incubated with counter cell lines (HCAEC and MCF10A) or other cancer and normal cell lines (Supplementary Fig. S4A–R). The fluorescence microscopy results matched the selectivity results obtained by flow cytometry, indicating that both aptamers are selective to their breast cancer cells using these two methods.

### MAMB1 and MAMA2 bind to their target proteins using Electrophoretic Mobility Shift Assay (EMSA)

Due to their high affinity and specificity (low K_d_ values) to their target cell lines, MAMB1 and MAMA2 aptamers were chosen to test their ability to interact with the target recombinant proteins that were used in the protein portion (MGB2 and MGB1) of the SELEX process. Utilizing EMSA with different concentrations of the recombinant proteins (0 to 220 nM) and a fixed concentration of the aptamer (600 nM), the results revealed that the aptamer-protein complex bands shifted upwards with increasing concentrations of the MGB2 and MGB1 proteins with their respective aptamers (Supplementary Fig. S5A and S5E).

MGB1-MAMA2 complex bands showed a higher mobility shift (Supplementary Fig. S5E) than MGB2-MAMB1 complex bands (60–220 nM). (Supplementary Fig. S5A). To confirm that the mobility shift was due to true binding of the aptamers to their target proteins, a dopamine DNA aptamer^45^ was incubated with the same concentrations of both proteins and used as a control. The control experiment results showed no significant shift of both aptamers when incubated with the control aptamer compared to the shifting of MAMB1 and MAMA2 aptamers with their target proteins (Supplementary Fig. S5B and S5F). Bovine Serum Albumin (BSA) protein was used as a second control, and EMSA results showed no significant shifting when MAMB1 and MAMA2 aptamers were incubated with BSA (Supplementary Fig. S5C and S5F). Both aptamer showed affinities in the nanomolar range, as indicated by plotting the different concentrations of aptamers (on the x-axis) against the shifting distance (cm) (Supplementary Fig. S5D and S5H).

### MAMB1 and MAMA2 targets on the surface of target breast cancer cells

In order to confirm that the selected aptamers are in fact binding to their anticipated biomarkers, a transfection approach was used that has been previously employed to identify aptamer target on the surface of cells after cell-SELEX^46,47^.

**Figure 6:**
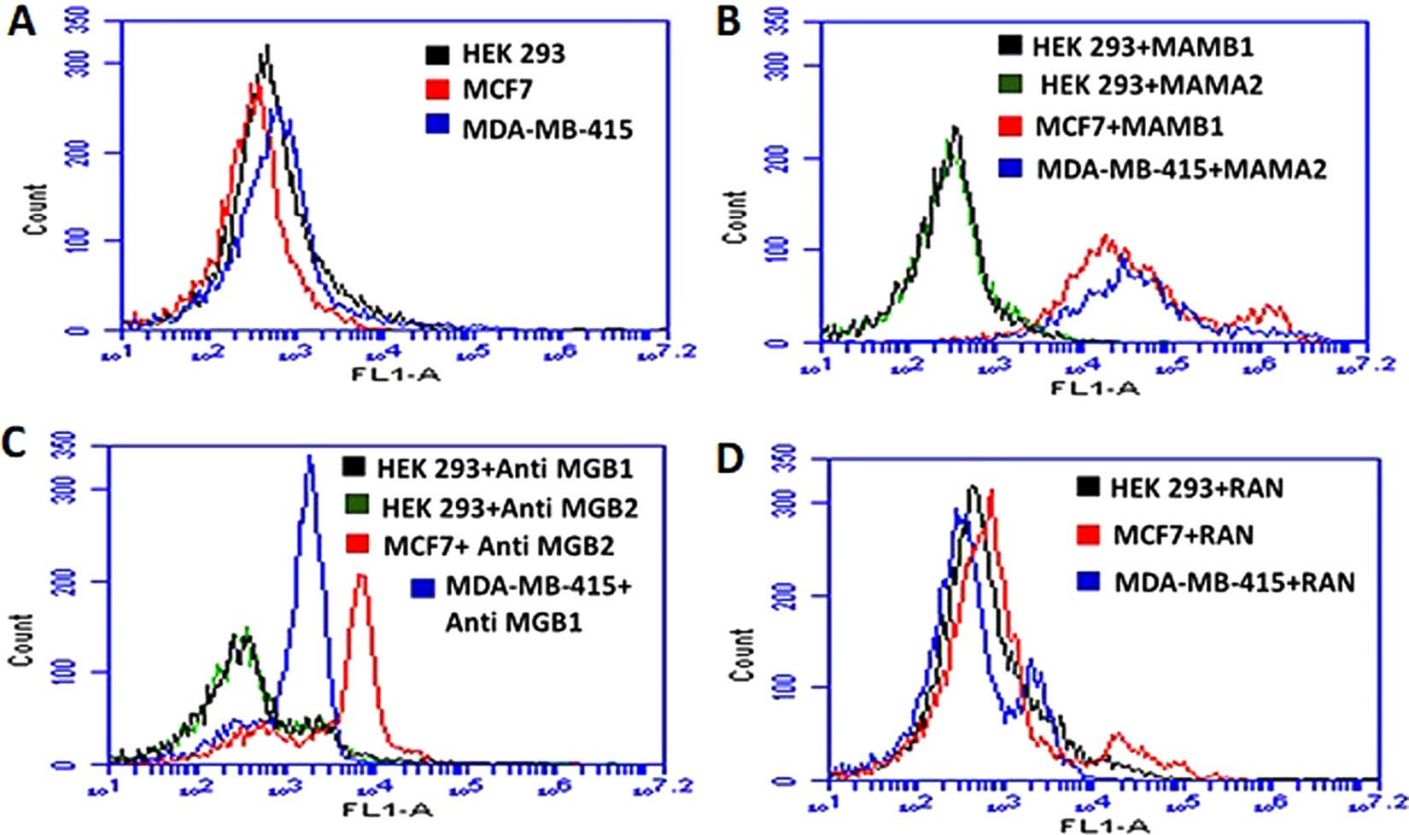
Binding of MAMB1, MAMA2 aptamers and MGB2, MGB1 antibodies to nontransfected HEK293, MCF7, and MDA-MB-415 cells. (**A**) Mean of fluorescence intensity of non-transfected HEK293 cells (black), MCF7 (red), and MDA-MB-415 (blue) without probes (cells only). (**B**) Mean of fluorescence intensity of non-transfected HEK293 cells binding to MAMB1 (black), MAMA2 (green), MCF7 binding to MAMB1 (red), and MDA-MB-415 binding to MAMA2 (blue). (**C**) Mean of fluorescence intensity of non-transfected HEK293 cells binding to anti-MGB1 antibody (black), anti MGB2 antibody (green), MCF7 binding to anti MGB2 antibody (red), and MDA-MB-415 binding to MGB1 antibody (blue). (**D**) Mean of fluorescence intensity of non-transfected HEK293 cells (black), MCF7 (red), and MDA-MB- 415 (blue) binding to a random sequence. Cells were incubated with their corresponding probes and 10000 events was counted by flow cytometry. Then data was analyzed using flow cytometry C6 sampler software.

**Figure 7:**
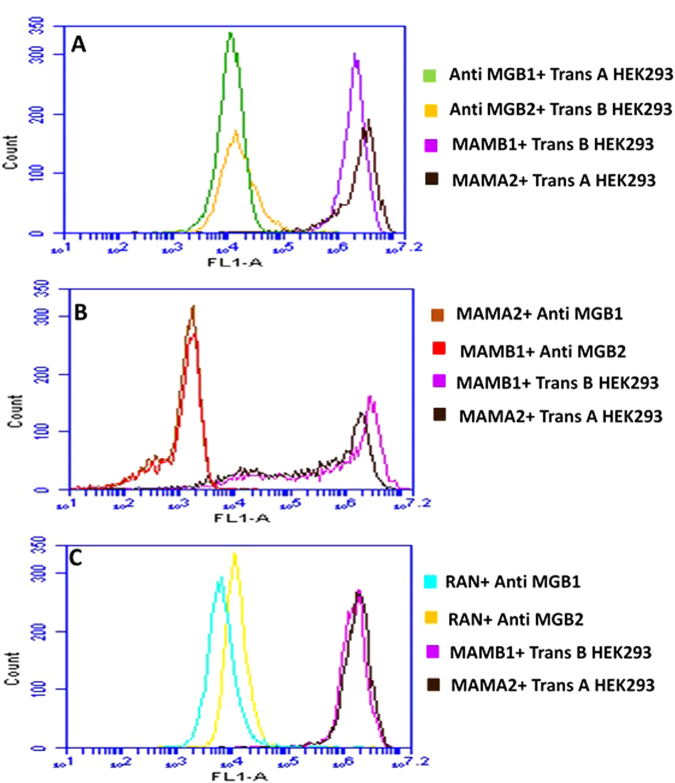
Competition binding assays of MAMB1, MAMA2 aptamers and anti MGB, anti MGB1 antibodies on transfected HEK293 cells with MGB2 and MGB1 plasmids. (**A**) Mean of fluorescence intensity of transfected HEK293 with MGB2 plasmid (Trans **B**) binding to anti MGB2 (orange) and MAMB1 (purple), and transfected HEK293 with MGB1 plasmid (Trans **A**) binding to anti MGB1 (green) and MAMA2 (brown). (**B**) Mean of fluorescence intensity of Trans B and Trans A HEK293 blocked with unlabeled MAMB1 and MAMA2 aptamers respectively, and probed with anti MGB2 (red) in the case of Trans B, and anti MGB1 (brown) in the case of Trans A. Mean of fluorescence intensity of Trans B and Trans A binding to MAMB1 (purple), and MAMA2 (brown) same as in A. (**C**) Mean of fluorescence intensity of Trans B and Trans A HEK293 blocked with random sequence (RAN) and probed with anti MGB2 (dark yellow) in the case of Trans B and anti MGB1 (light blue) in the case of Trans A. Mean of fluorescence intensity of Trans B and Trans A binding to MAMB1 (purple) and MAMA2 (brown) same as in A. Cells were incubated with their corresponding probes and 10000 events was counted by flow cytometry. Then data was analyzed using flow cytometry C6 sampler software.

HEK293 cells were chosen to investigate the targets of MAMB1 and MAMA2. HEK293 are derived from human embryonic kidney cells and are widely used as a host for gene expression^48^. Therefore, they were used as a host for MGB2 and MGB1 plasmids. HEK293 was proven not to express MGB2 and MGB1 proteins compared to target breast cancer cells (MCF7 and MDA-MB-415) using ELISA kits (Supplementary Fig. S6). Moreover, transfected HEK293 cells were used in the competition studies using monoclonal anti-MGB2 and anti-MGB1, MAMB1 and MAMA2. The results showed that both aptamers and antibodies did not bind to non-transfected HEK293 cells, but showed binding to MCF7 and MDA-MB-415 cells (Fig. 6). This binding was 20-fold more to transfected HEK293 when incubated with MAMB1 and MAMA2 aptamers, and 10-fold more when incubated with MGB2 and MGB1 antibodies (Fig. 7A).

It was also observed that both aptamers and antibodies compete on the same binding sites of MGB2 and MGB1 overexpressed proteins on the surface of transfected HEK293 cells (Fig. 7B). When transfected HEK293 cells (with either MGB2- or MGB1-expressing plasmids) were blocked with unlabeled MAMB1 and MAMA2 aptamers and then probed with anti-MGB2 and anti-MGB1 respectively, the mean of fluorescence intensity decreased by almost 10-fold (Fig. 7B). Blocking with a random sequence restored the binding of anti-MGB2 to transfected MGB2 HEK293 cells (Fig. 7C dark yellow) same as the binding seen in Fig. 7A, suggesting that both MAMB1 and anti-MGB2 are competing for the same binding sites. This binding is specific as it was restored when a random sequence was used for blocking (Fig. 7C). A small shift in the mean of fluorescence intensity of anti-MGB1 binding to transfected MGB1 HEK 293 cells was seen when the same random sequence was used to blocked the binding sites of MGB1 on transfected MGB1 HEK293 cells (Fig. 7C, light blue). This could be non-specific binding of the random sequence to MGB1 sites on transfected MGB1 HEK293 cells. Other random sequences could be used in the future to restore the original binding seen in Fig. 7A.

pUC19-transfected HEK293 results showed no binding to MAMB1, MAMA2, anti MGB2, and anti-MGB1 probes (Supplementary Fig. S7). Moreover, no binding was seen when pUC19-transfected HEK293 cells were blocked with either unlabeled MAMB1 and MAMB1 aptamers or with a random sequence. pUC19 did not contain either MGB2 or MGB1 genes and therefore MGB2 and MGB1 were not expressed on the surface of HEK293 cells. The mean of fluorescence intensity of pUC19 transfected HEK293 cells was close to the one for non-transfected HEK293 cells (Fig. 6A).

**Figure 8:**
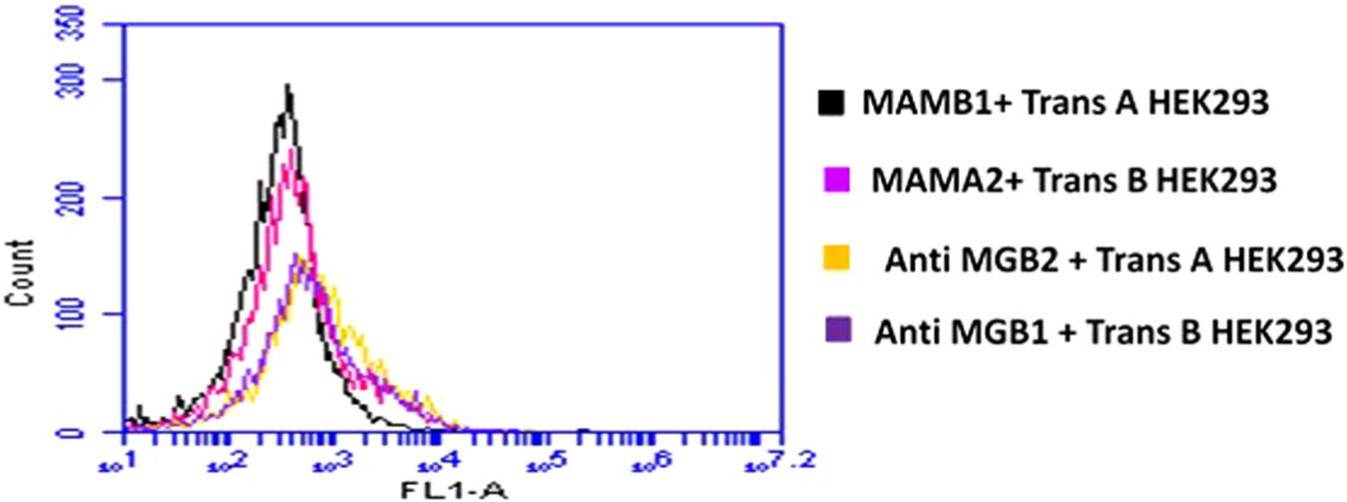
Selectivity test performed on transfected HEK293 cells with MGB2 and MGB1 plasmids. The figure shows the mean of fluorescence intensity of MAMB1 (black), MAMA2 (pink), anti MGB2 (dark yellow), and anti MGB1 (purple) binding to Trans A and Trans B HEK293 cells respectively. Cells were incubated with their counter probes transfected HEK293, and 10000 events was counted by flow cytometry. Then data was analyzed using flow cytometry C6 sampler software.

To investigate the selectivity of both aptamers and antibodies, the binding of MAMB1 and MAMA2 to transfected MGB1 and transfected MGB2 HEK293, respectively, was tested (Fig. 8). The results showed that MAMB1 did not bind to transfected MGB1 HEK293 cells (Fig. 8, black), and the same was observed for MAMA2 aptamer (Fig. 8, pink). This was expected as steps were taken in SELEX to eliminate sequences that could bind to both MGB2 and MGB1 proteins together. The MGB2 and anti-MGB1 to transfected MGB1 (Fig. 8, dark yellow) and transfected MGB2 HEK293 cells (Fig. 8, purple) respectively, showed a small shift in the mean of fluorescence intensity. This could be as a result of non-specific binding of both antibodies to their counter proteins. These results indicate that both MAMB1 and MAMA2 aptamers are more selective than their antibodies counterparts. In summary, our aptamer–antibody competition binding assay results confirmed that MGB2 and MGB1 proteins are the targets of MAMB1 and MAMA2 aptamers respectively.

### Binding of MAMB1 and MAMA2 to breast cancer cells in plasma and whole blood lysate

The ability of MAMB1 and MAMA2 to bind selectively to breast cancer cells in plasma and whole blood lysate was investigated using peripheral blood mononuclear cells (PBMC), which represent normal peripheral blood cells and include mainly lymphocytes (T cells, B cells, natural killer cells) and monocytes. These cells were used as the control (i.e. non-cancerous cells). MCF7 and MDA-MB-415 were used as the target breast cancer cells for MAMB1 and MAMA2 aptamers, respectively.

**Figure 9:**
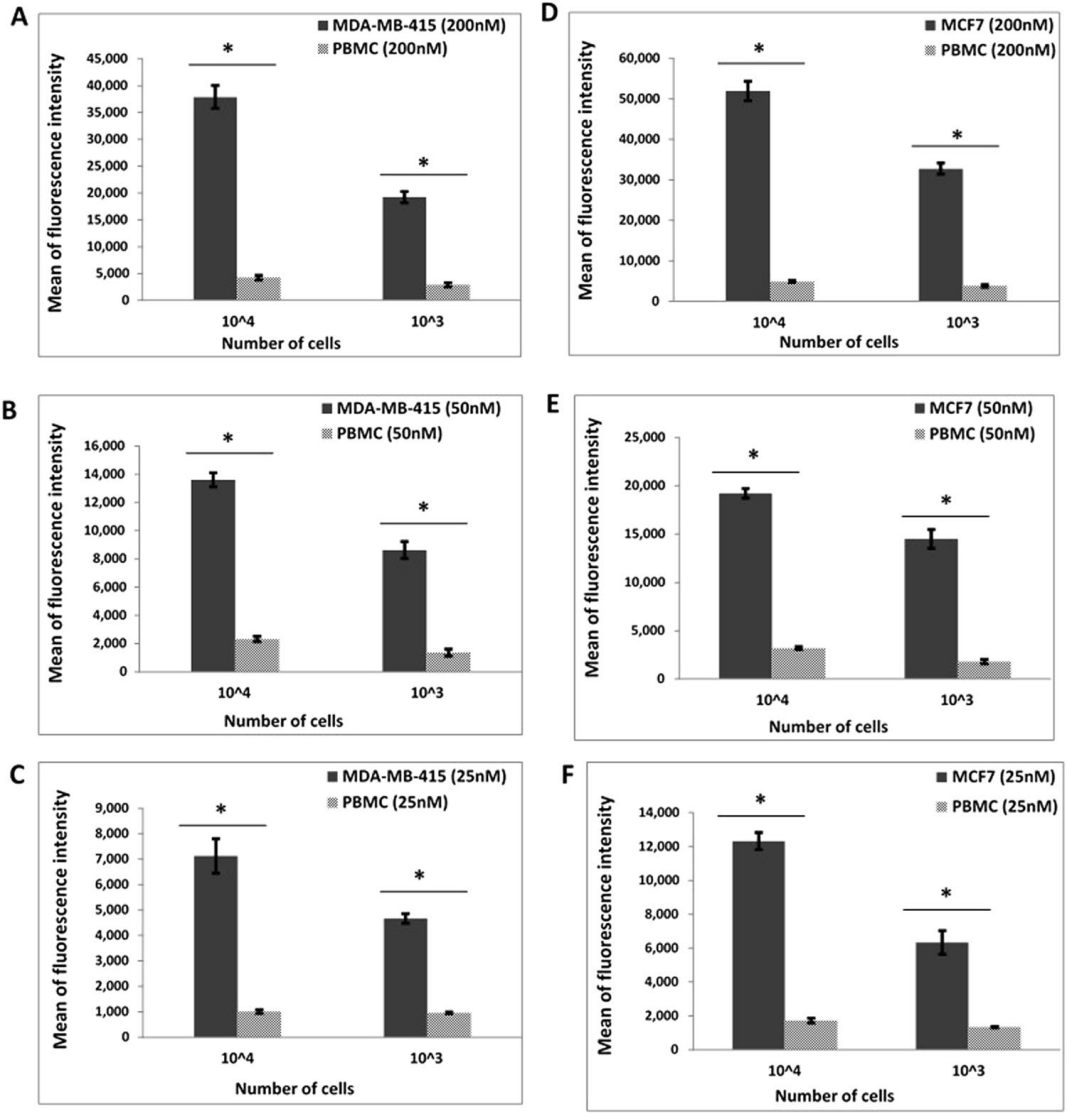
Binding of MAMB1 and MAMA2 aptamers to their breast cancer cells in plasma by flow cytometry. (A-C) Mean of fluorescence intensity of MDA-MB-415 cancer cells and PBMC normal cells at 200nM (**A**), 50nM (**B**), and 25nM (**C)** concentrations of MAMA2 aptamer. (**D–F**) Mean of fluorescence intensity of MCF7 cancer cells and PBMC normal cells at 200nM (**D**), 50nM (**E**), and 25nM (**F**) concentrations of MAMB1 aptamer. Two cell numbers from both cancer and normal cells were used: 10^4^ and 10^3^ cells. Values are shown as means ± S.E.M. of three trials. The statistical significance was determined by one way ANOVA followed by Fisher LSD multiple tests using SPSS software (SPSS, version 23). P Values less than 0.05 were considered to be significantly different.

The human plasma used in this study was prepared from pooled human blood (Sigma Aldrich, Canada) and contained active clotting factors. The ability of MAMB1 and MAMA2 to bind to their target breast cancer cells in plasma was first investigated. The study aimed to provide a proof-of-concept, demonstrating the selective binding of MAMB1 and MAMA2 aptamers to their specific cell lines, in a series of different environments, starting with least complex to the most complex. Starting with plasma, as the least complex environment, MAMB1 and MAMA2 6-FAM labeled aptamers discriminate breast cancer cells against normal blood cells (PBMC) (spiked into the plasma as described in the methods section), as shown by the difference in mean of fluorescence intensity (Fig. 9). MAMB1 and MAMA2 behaved the same and showed significant binding (P < 0.05) to their target breast cancer cells at different cell numbers (1 × 10^4^ and 1 × 10^3^ cells) and different aptamer concentrations (25, 50, 200 nM). MAMB1 had a higher affinity to its target breast cancer cell line (MCF7) than MAMA2.

**Figure 10:**
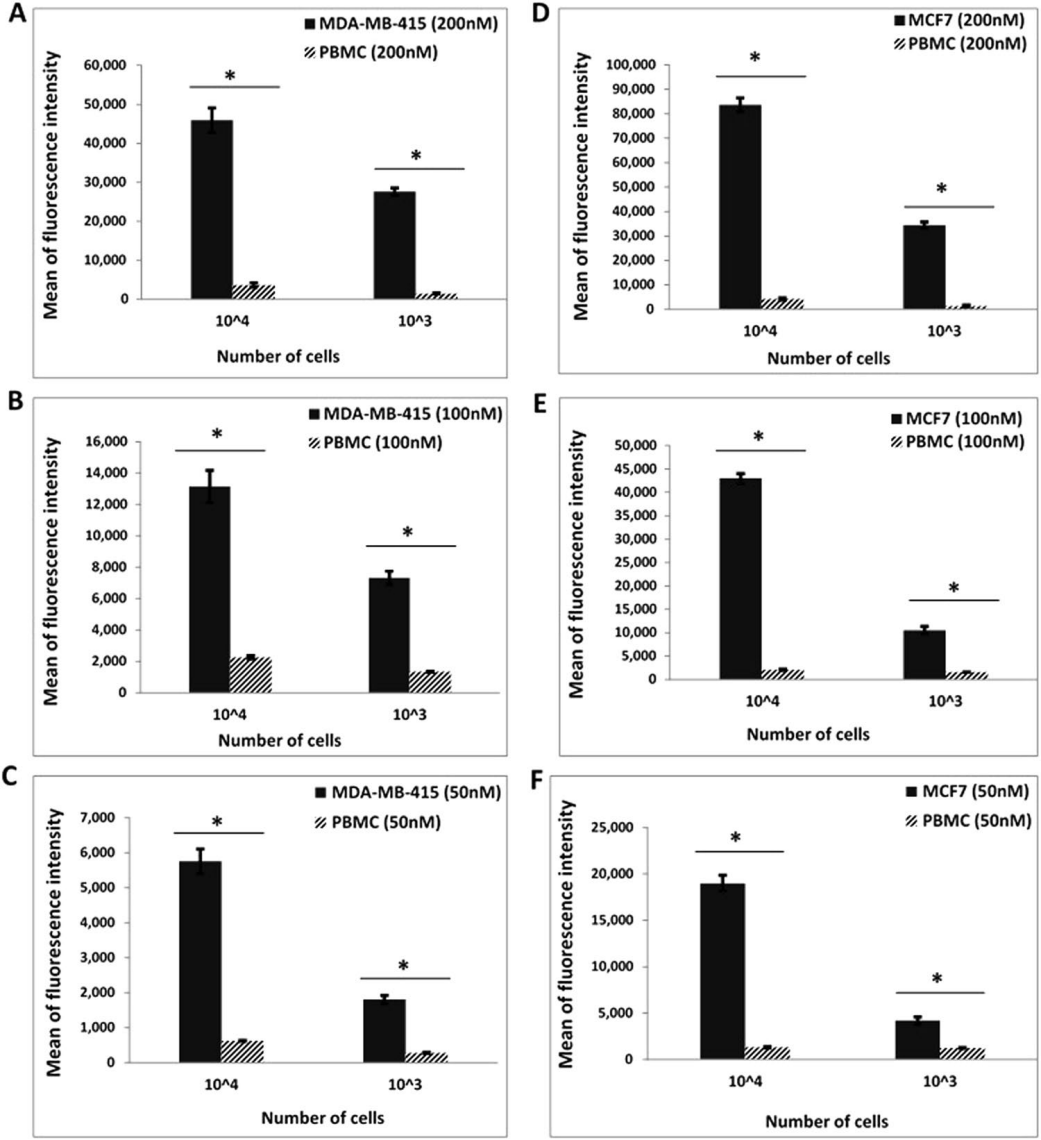
Binding of MAMB1 and MAMA2 aptamers to their breast cancer cells in whole Blood lysate by flow cytometry. (**A**–**C**) Mean of fluorescence intensity of MDA-MB-415 cancer cells and PBMC normal cells at 200 nM (**A)**, 100 nM (**B**) and 50 nM (**C**) concentrations of MAMA2 aptamer. (**D–F**) Mean of fluorescence intensity of MCF7 cancer cells and PBMC normal cells at 200nM (**D**), 100nM (**E**) and 50nM (**F**) concentrations of MAMB1 aptamer. Two cell numbers from both cancer and normal cells were used: 10^4^ and 10^3^ cells. Values are shown as means ± S.E.M. of three trials. The statistical significance was determined by one way ANOVA followed by Fisher LSD multiple tests using SPSS software (SPSS, version 23). P Values less than 0.05 were considered to be significantly different.

MGB1 and MGB2 were previously reported as biomarkers for CTCs in the blood of breast cancer patients^24–28^. As such, blood was selected as the second most complex environment to investigate the binding of MAMB1 and MAMA2 aptamers to their breast cancer cells targets, in comparison to PBMC cells (cells were added to the blood lysate as described in the method section). The whole blood lysate experimental results showed that both aptamers bind specifically to their target breast cancer cells (Fig. 10). The mean of fluorescence intensity of cancer cells bound to both aptamers was significantly increasing (p < 0.05) with increasing the aptamer concentration (50, 100 nM) and number of cells (10^3^, 10^4^) for both cell lines (Fig. 10).

Our whole blood lysate might include nuclease enzymes that could degrade aptamers and interfere with their binding to breast cancer cells. It has been reported that chemical modification of aptamers could improve their binding capability and make them more resistant to nucleases^49,50^. In the present study, both aptamers were chemically unmodified, with the only modification being the labelling with 6-FAM for both. MAMA2 showed less affinity towards its target, compared to MAMB1, in both plasma and whole blood lysate and it may require modification to improve its binding affinity. Nevertheless, fluorescence intensity values for both aptamers binding in whole blood lysate was comparable to that seen in plasma, indicative that, overall, MAMB1 and MAMA2 aptamers showed great potential as detection tools for breast cancer cells in the plasma and whole blood lysate. To date, no study has been done to detect MGB1 and MGB2 in the blood or plasma of breast cancer patients, based on cell recognition, using flow cytometry.

### Binding of MAMB1 and MAMA2 to spiked breast cancer cells in whole blood lysate

**Figure 11:**
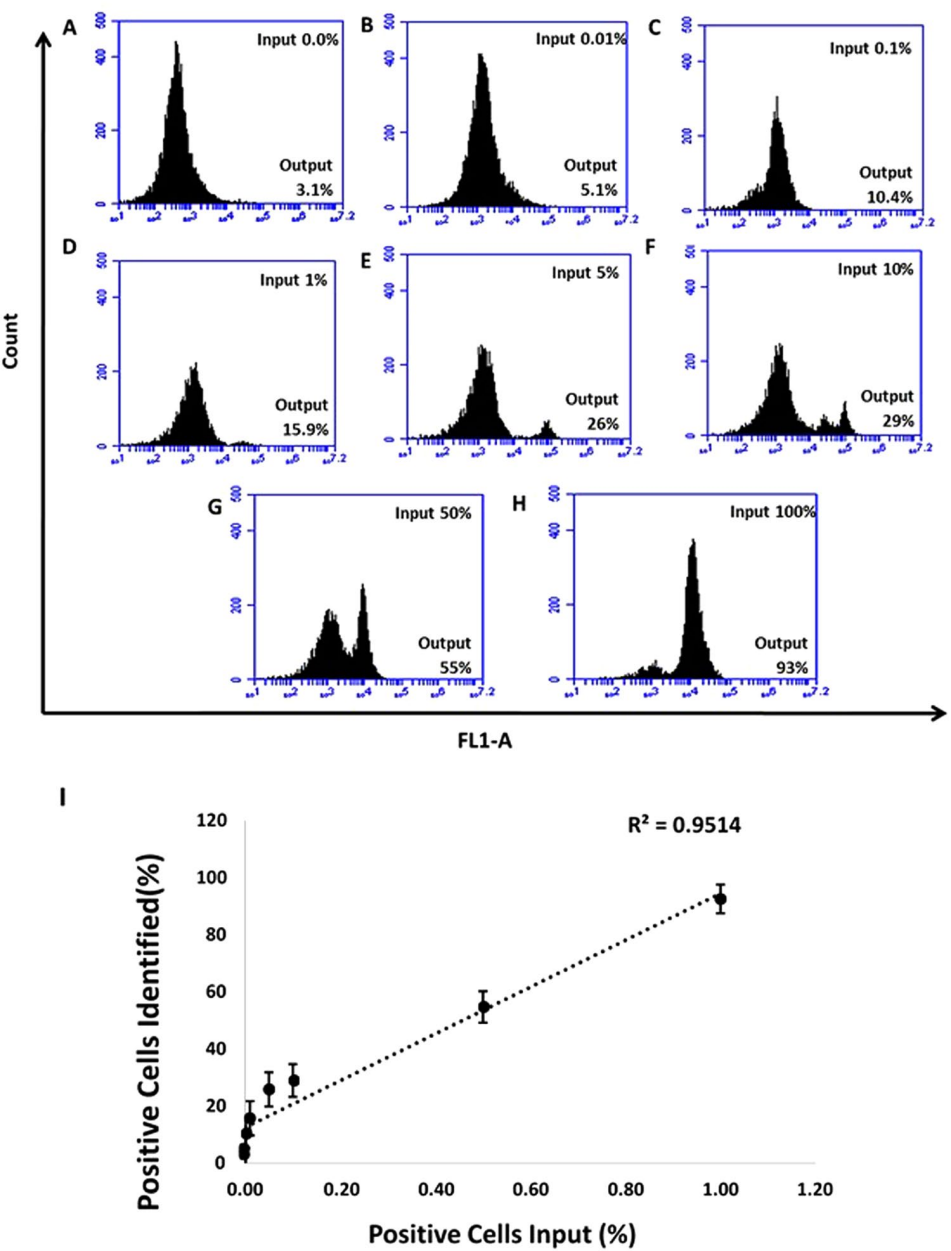
Selective recognition of MCF7 cells in mixed cell samples with the 6-FAM labelled MAMB1 aptamer in whole blood lysate. Cell mixture samples containing MCF7 and PBMC were prepared in different percentages of MCF7 ((**A–G**) 0%, 0.01%, 0.1%, 1%, 5%, 10%, 50%, and 100%). (**I**) plot of the percentage of cancer cells (MCF7) spiked (X-axis) and the percentage of positive cells identified (Y-axis). Values are shown as means ± S.E.M. of three trials. The statistical significance was determined by one way ANOVA followed by Fisher LSD multiple tests using SPSS software (SPSS, version 23). P Values less than 0.05 were considered to be significantly different.

**Figure 12:**
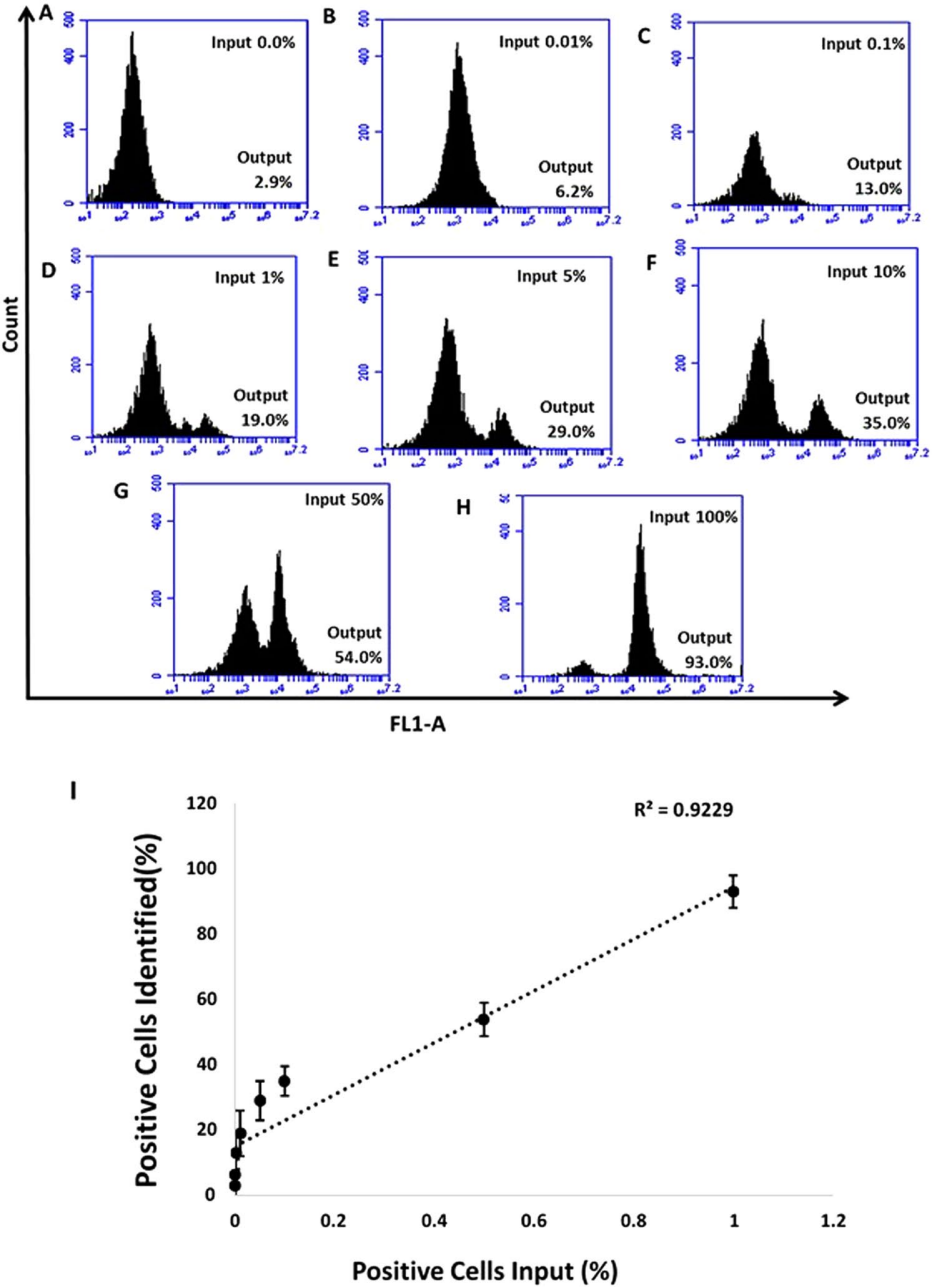
Selective recognition of MDA-MB-415 cells in mixed cell samples with the 6-FAM labelled MAMA2 aptamer in whole blood lysate. Cell mixture samples containing MDA-MB-415 and PBMC were prepared in different percentages of MDA-MB-415 ((**A**–**G**) 0%, 0.01%, 0.1%, 1%, 5%, 10%, 50%, and 100%). (**I**) Plot of the percentage of cancer cells (MDA-MB-415) spiked (X-axis) and the percentage of positive cells identified by the MAMA2 aptamer (Y-axis). Values are shown as means ± S.E.M. of three trials. The statistical significance was determined by one way ANOVA followed by Fisher LSD multiple tests using SPSS software (SPSS, version 23). P Values less than 0.05 were considered to be significantly different.

The selective binding of MAMB1 and MAMA2 aptamers were further tested in an environment in which MCF7 and MDA-MB-415 (positive cells) were spiked in different percentages (0, 0.01, 0.1, 1, 5, 10, 50, and 100) in whole blood lysate containing PBMC cells (that were added to the blood initially), and then incubated with 200 nM of MAMB1 and MAMA2 aptamers, respectively. The results obtained showed that the percentages of positive cells (output cells recognized by 6-FAM labeled aptamers) identified by the flow, increased with increasing number of spiked breast cancer cells (percentage of input cells) from 3.1% (for PBMC cells in blood lysate) (Fig. 11A) to 93.0% (for MCF7 cells in blood lysate) (Fig. 11H) for MAMB1 aptamers, and from 2.9% (for PBMC cells in blood lysate) (Fig. 12A) to 93.0% (for MDA-MB-415 in blood lysate) (Fig. 12H) for MAMA2 aptamer. The relationship between the percentage of spiked cancer cells (X-axis) and the percentage of positive cells identified (Y-axis) was linear (Figs 8I and 9I). MAMB1 and MAMA2 aptamers showed significant binding (P < 0.05) to spiked MCF7 and MDA-MB-415 cancer cells respectively in whole blood lysate containing PBMC cells at 5, 10, 50, and 100%. It has been reported that CTCs were detected at 10% using flow cytometry^51^. In our study, we significantly (P < 0.05) detected CTCs at 5%. The detection at 0.01, 0.1, and 1% was approaching significant (P = 0.07). Indicating that both aptamers could be used in a platform to detect CTCs from breast cancer.

## Conclusion

In summary, a hybrid-SELEX approach was successfully used to select aptamers (MAMB1 and MAMA2) that target MGB2 and MGB1 biomarkers expressed on the surface of MCF7 and MDA-MB-415 cells, respectively. The target of these aptamers was confirmed by EMSA with their respective recombinant proteins, and by flow cytometry after transfection of MGB2 and MGB1 plasmids into HEK293 cells. The selected aptamers showed high affinity to their targets on the surface of the cells (K_d_ of low nanomolar range values), as well as to their free proteins. Both aptamers showed binding with high specificity and selectivity to breast cancer cells (as compared to normal breast cells, other cancer cell lines and non-cancer cells) using flow cytometry and fluorescence microscopy. Both aptamers were able to effectively discriminate breast cancer cells from normal blood cells. Moreover, the potential of both aptamers to bind to spiked breast cancer cells in whole blood lysate revealed the possibility of using both aptamers as CTC markers for the early detection of breast cancer.

## Materials and Methods

### Cell lines and cell culture

All cell lines were purchased from American Type Culture Collection (ATCC) unless otherwise indicated. MCF-7, (breast adenocarcinoma, derived from metastatic site: pleural effusion), MDA-MB-415 (breast adenocarcinoma, derived from metastatic site: pleural effusion), were used as the target cell lines. MCF-10A (derived from mammary gland; breast) and HCAEC (Human Coronary Artery Endothelial Cells; primary cells) cell lines were both used as counter selection lines. The following cells lines were used in specificity tests: HepG2 (hepatocellular carcinoma), HeLa (human cervical carcinoma), HEK293 (human embryonic kidney), WI-38 (human lung normal) and MDA-MB-231 (breast adenocarcinoma, derived from metastatic site: pleural effusion) which was obtained as a frozen stock from Dr. Christine Pratt (Cellular and Molecular Medicine, University of Ottawa, Canada). Human Primary Peripheral Blood Mononuclear Cells (PBMC) (normal) were used in the blood and plasma experiments.

MCF7, MDA-MB-415, MDA-MB-231, HepG2, HeLa, HEK293 cells were cultured in DMEM. Human recombinant insulin (0.01 mg/ml) was added as supplement to only MCF7 and MDA-MB-415. WI-38 cells were cultured in EMEM medium. Fetal bovine serum (FBS; 10%) was added to the media of all the above cell lines. MCF10A cells were cultured in Mammalian Epithelial Cell Growth Medium kit (Lonza). HCAEC were cultured in MV2 basal medium kit (PromoCell). PBMC cells were cultured in Hank’s balanced salt solution. All cells were cultured in a 5% CO_2_, 95% humidified chamber at 37 °C.

### DNA Synthesis

The ssDNA library was prepared with a 24 nucleotide randomized sequence (N), flanked by two 20-nucleotide primer binding sites. ssDNA library: 5′-CATGCTTACCTATAGTGAAC (N24) CTTTGAGAACTGACTCTAC-3′, the forward primer was labelled on the 5′ end with fluorescein to monitor the library enrichment during SELEX (5′-(6-FAM)-CATGCTTACCTATAGTGAAC-3′). Twenty adenine nucleotides and a hexaethyleneglycol (HGE) linker were added to the reverse primer at the 5′ end (5′-(A20)-HEG-GTATGAGTCAGTTCTCAAAG-3′) to be able to separate the forward from reverse strands by denaturing PAGE (12% at 250 V, 3 hrs).6-FAM was added to the five prime ends to all the synthesized aptamers. The MerMade 6 automated DNA synthesizer (Bioautomation, USA) was used to synthesize all DNA sequences and the library.

### Hybrid-SELEX approach

Purified GST tagged recombinant MGB2 protein (target protein), and purified GST protein (counter selection target) were conjugated to glutathione-agarose beads by incubating 16 µg of GST protein and 9 µg of MGB2 in 100 µL of the beads in binding buffer (150 mM Tris, 150 mM NaCl, 5 mM KCl, 5 mM MgCl_2_, pH 7.4) at RT for 30 min. Beads were then centrifuged (21000 × g for 2 min) and supernatant was kept for protein measurement at 280 nm. SELEX was initiated with a counter selection round by incubating GST-conjugated beads (100 µL) with a ssDNA library (5 nmol) which was heated at 95 °C for 5 min and then gradually cooled to 4 °C for 10 min and another 10 min at room temperature. The mixture was then incubated at room temperature in binding buffer for 60 min. The supernatant was then subjected to further positive selection by incubating it with purified GST-tagged MGB2 protein-conjugated beads (beads after conjugation above) for 30 min at room temperature. A partitioning step took place by washing the beads with washing buffer (same as the binding buffer). The protein-DNA complex was then eluted from the beads (5 times 100 µL each) using elution buffer (7 M urea, 5 mM EDTA) heated to 95 °C, followed by phenol-chloroform extraction and ethanol precipitation^52^. The eluted DNA was then amplified by PCR using the following conditions: 95 °C for 30 s, 58 °C for 30 s, and 72 °C for 30 s for 25 cycles, followed by extension step at 72 °C for 10 min. The SELEX was repeated for 21 rounds and the counter selection step was repeated every round, along the positive selection step. MGB1 protein-conjugated beads were then used as a counter selection step instead of GST-conjugated beads in rounds 16, 17 and 18 to remove aptamers targeting MGB1 protein, because of their high similarity, resulting in the separation of the initial ssDNA library into two halves. Another separate SELEX for MGB1 was performed in a manner similar to that for MGB2 SELEX, using four rounds of selection and GST-conjugated beads as the counter selection in every round (Fig. 1). The stringency of MGB2 and MGB1 selection was increased by decreasing the concentration of the amplified ssDNA library (from 5 nmol to 100 pmol in the final pools) and the time of incubation for the positive targets (from 30 min to 15 min), and increasing the washing times (from 2 to 5 times) and volume (from 100 to 500 µL).

In order to obtain aptamers that bound to MGB2 and MGB1 on the surface of the cells, the final enriched DNA libraries resulting from protein SELEX were used as the initial pools for cell-SELEX. In cell-SELEX, two counter selection steps took place in which 2 × 10^6^ cells of MCF-10A and HCAEC were incubated with 100 pmol of the enriched libraries (after heating and cooling to room temperature) in binding buffer (4.5 g/L glucose, 5 mM MgCl_2_, 2 mg/mL BSA, 0.2 mg/mL yeast tRNA, 200 µL of 10% FBS in PBS, pH 7.4) for 1 hour at 4 °C. The supernatants of MGB2 and MGB1 libraries were then incubated with 1 × 10^6^ cells of MCF7 and MDA-MB-415, respectively in binding buffer for 30 min at 4 °C. Cells were then washed with washing buffer (4.5 g/L glucose, 5 mM MgCl_2_ in PBS, pH 7.4) to remove any unbound sequences. Bound DNA was then eluted in washing buffer by heating at 95 °C for 5 min. The supernatant was then collected and amplified by PCR using the same conditions as in the protein-SELEX, but the number of cycles was reduced to 20. The cell-SELEX was repeated for 7 rounds for both MCF7 (MGB2) and MDA-MB-415 (MGB1) cell lines. The enrichment of both libraries was gradually increased during SELEX by reducing the amount of both pools (from 100 to 50 pmol), the incubation time for the target cells (from 30 to 15 min), and the target cell number (from 1 × 10^6^ to 1 × 10^5^ cells) and by increasing the number of washes (from three to six). To eliminate the off-target binding aptamers that might result from cell-SELEX, an additional 2 rounds of purified protein-SELEX were performed to further focus the ssDNA pools specifically toward MGB2 and MGB1 (Fig. 1).

### Monitoring the enrichment of hybrid SELEX

MCF7, MDA-MB-415, HCAEC, and MCF10A cells (2 × 10^5^ cells) were incubated with 200 nM of ssDNA libraries and 0.3 mg/mL of ssDNA from salmon sperm, 0.5 mg/mL bovine serum albumin (BSA) for 30 min at 4 °C. Then cells were washed and re-suspended in 250 µL of binding buffer. Fluorescence intensity was analyzed from a minimum of 10000 cells using a BD Accuri C6 benchtop flow cytometer and C6 sampler software (BD, USA). Round 0 ssDNA library was used as the negative control and treated same way as the ssDNA libraries from cell SELEX.

### High throughput sequencing

High throughput sequencing (HTS) was performed using Illumina sequencing. Briefly, the enriched ssDNA pools from different rounds of selection (protein and cell-SELEX) for MGB2 (R0, R16, R16 counter selection, R30, R30 counter selection) and MGB1 (R17 counter selection of MGB1, R4, R5, R7, R10, R11, R12, R13 counter selection) SELEX were PCR amplified using Illumina special adaptors (TruSeq primers) to a minimum number of cycles (17 cycles). PCR products were purified using PAGE and the DNA quantified using a Nanodrop spectrophotometer (Thermo Fisher, Canada). All MGB1 amplified pools were combined and all MGB2 amplified pools were combined separately to provide a total of 75 ng of DNA in each pool. Amplified pools were then sent to Genome Quebec Facility (Quebec, Canada) to be sequenced using Illumina HiSeq sequencing platform. AptaCluster was used as the software to analyze the sequencing data and RNAstructure software (Mathews Lab RNAstructure) was used to predict the secondary structure of the candidate sequences.

### Assessment of the binding affinity and selectivity (cell type)

All selected aptamers were evaluated for their binding affinity in a screening test by flow cytometry in which the fluorescence intensity was determined for all selected aptamers against positive and counter selection cell lines. Second, aptamers that showed high binding affinity to positive cell lines and minimum non-specific binding to counter cell lines were chose to determine their apparent dissociation constants (K_d_) using different concentrations of the aptamers (0, 5, 10, 40, 60, 100, 140, 160, 180, 200 nM) that were incubated with fixed MCF7 and MDA-MB-415 cells (2 × 10^5^) in 200 µL total volume of binding buffer. Round 0 was used as the control and incubated with the same concentrations of aptamers.

To assess the selectivity of the aptamers, the remaining aptamers were incubated with all other cell lines (2 × 10^5^ fixed cells in 200 µL in binding buffer) mentioned above and were subjected to determination of the binding affinity by flow cytometry.

### Assessment of binding (recombinant protein)

Electrophoretic mobility shift assay (EMSA) was used to investigate the binding of selected aptamers to their target recombinant proteins (MGB2 and MGB1). Briefly, different concentrations of target proteins (0 to 220 nM) were incubated with a fixed aptamer concentration (600 nM) in 50 mM Tris-HCl, 10 mM reduced glutathione, pH 8.0 for 1 hr at 37 °C. Protein-aptamer complex samples were then loaded to 8% non-denaturing polyacrylamide gel and allowed to run for 3 hrs at 160 V at room temperature. Gels were then visualized using an Alpha Innotech gel imager documentation system (USA). ssDNA aptamer against dopamine (60 bases^45^) and BSA protein were used as controls by incubating them with increasing concentrations of the same target proteins and treated the same way as the experimental aptamers.

### Binding of MAMB1 and MAMA2 to their target breast cancer cells by fluorescence microscopy

The binding of MAMB1 and MAMA2 to their target breast cancer cells was further by fluorescence microscopy. Briefly, cells (5 × 10^5^) were plated on coverslips in 35 mm tissue culture dishes and let to grow for 24 hours, cells then were washed with PBS and fixed in 4% formaldehyde solution for 15 min at RT. Aptamers were added for 30 min at RT at concentration of 300 nM in binding buffer. Cells then were washed three times with cold PBS, then 4′,6-diamidino-2-phenylindole (DAPI) (10 mg/mL diluted 1:20,000) was added for 5 min. Cells then were washed three times each 5 min using deionized water. Then coverslips were mounted on microscope slides using VECTASHIELD mounting medium (Vector laboratories, USA) and imaged using ZOE™ fluorescent cell Imager (Bio-Rad, Canada) using the green and blue channels.

MCF7 and MDA-MB-415 were used as the target breast cancer cells lines for MAMB1 and MAMA2 respectively, HCAEC, MCF10A, HEK293, WI38, MDA-MB-231 and HepG2 cell lines were used to test the selectivity of both aptamers. Random sequence (dopamine aptamer) was used as control, which was incubated same way as the aptamers with each cell line and imaged using fluorescence microscopy.

### Binding of MAMB1 and MAMA2 to their target breast cancer cell lines in plasma and whole blood lysate

Lyophilized human plasma (Sigma Aldrich) was dissolved in 5 mL of deionized water on a shaker for 2 hrs at room temperature. According to the source of lyophilized human blood sample, the blood sample was blood obtained from a normal human donor that was freeze dried. All the components of normal blood were there including cells and plasma proteins (such as albumins, globulins, fibrinogen) but the cells, in this case, are lysed (dead). The sample was dissolved in 3 mL of deionized water on a shaker for 3 hrs at room temperature. A smear of the reconstituted blood was made to check for the existence of cells. Blood samples were then lysed using RBC lysis buffer (154 mM NH_4_Cl, 5.7 mM K_2_HPO_4_, 0.1 mM EDTA).

MCF7, MDA-MB-415, and PBMC cells were harvested and fixed in 4% formaldehyde solution in PBS for 15 min at room temperature. Cells then were centrifuged at 1,000 × g for 5 min, and re-suspended in human whole blood lysate and human plasma prepared previously (1 mL each). The total number of cells from each cell line was 1 × 10^7^. Then cell-whole blood lysate and cell-plasma suspensions from all three cell lines was used as stocks to prepare two numbers of cells which are 1 × 10^3^ and 1 × 10^4^ cells from each cell line. For whole blood lysate experiments, MAMB1 and MAMA2 aptamers (heated at 95 °C and gradually cooled down 10 min at 4 °C and 10 min at room temperature prior to use) were incubated with the different numbers of cells from each corresponding cell line (MAMB1 with MCF7 and MAMA2 with MDA-MB-415) at concentrations of 200, 100, and 50 nM final concentrations and 250 µL final volume at 4 °C for 15 min. The plasma experiments had the same procedure applied as mentioned above, but the final concentrations of the aptamers were 200, 50, and 25 nM. PBMC cells were used as the control for both plasma and whole blood lysate experiments and they were treated same way as the target cells. After incubation, all samples were subjected to flow cytometry analysis.

### Binding of MAMB1 and MAMA2 to their spiked breast cancer cells in whole blood lysate

Breast cancer cells were harvested and fixed same way as in the plasma and whole blood lysate experiments. MCF7 and MDA-MB-415 cells were then spiked in different percentages in PBMC cells-whole blood lysate suspension to a total number of 1 × 10^4^ cells. The percentages of spiked cancer cells, for each type of cells, were 0.01, 0.1, 1, 5, 10, 50, and 100%. MAMB1 and MAMA2 aptamers (200 nM each) were incubated with their corresponding spiked breast cancer cells in whole blood lysate in a total volume of 250 µL for 15 min at room temperature. Cells were then used for flow cytometry analysis.

### Aptamers cells binding studies to identify the targets on the surface of breast cancer cells

HEK293 cells were chosen to perform the aptamers cells binding studies. The expression levels of both proteins MGB2 and MGB1 was evaluated using ELISA kits for both proteins. MGB2 and MGB1 plasmids were then used to transfect HEK293 cells. Briefly, HEK293 cells (5 × 10^5^) were plated in 60 mm tissue culture dishes and left to grow for 24 hours in the incubator (37 °C and 5% CO_2_). Then, next day the old DMEM medium was replaced with new one (5 mL) and HEK 293 cells were left in the incubator for 30 min. The DNA (plasmids) were prepared for transfection; 10 µg of each purified MGB1 and MGB2 plasmids were added to 250 µL of Opti-MEM media, then 50 µL (final concentration of 5 µg/mL) of polyethylenimine (PEI) was added to 250 µL of Opti-MEM media in a separate tube. Then PEI was added to the DNA (plasmids) and incubated for 15 min at RT. The PEI-DNA was mixed by vortex every 3 min for 5 sec. After that, the PEI-DNA complex was added to the cells gradually drop by drop, and then HEK293 cells were left to grow for 48 hours. pUC19 empty vector was used as control. green fluorescence protein (GFP) was used to validate the transfection efficiency. Both pUC19 and GFP plasmids were transfected using the same amount of DNA as MGB2 and MGB1 plasmids under the same conditions and time. After 48 hours, HEK293 cells were harvested and fixed with 4% formaldehyde for 15 min at RT. Then washed and re-suspend in PBS to be used in flow cytometry analysis.

For aptamers antibodies competition studies, anti MGB2 and anti MGB1 monoclonal antibodies, goat anti-mouse FITC labelled secondary antibody, 6-FAM-labelled and unlabeled MAMB1 and MAMA2 aptamers, non-transfected and transfected HEK293 cells as well as MCF7 and MDA-MB-415 were used in competition study. Briefly, 5 × 10^5^ fixed cells of each cell line mentioned above was added to 500 µL of 5 mM MgCl_2_ in PBS, then competition studies were performed as the following. 1): non-transfected HEK293, MCF7 and MDA-MB-415 cells were incubated with 300 nM of 6-FAM labelled aptamers (MAMB1 with MCF7, MAMA2 with MDA-MB-415, and each of them with HEK293) or a control random sequence for 30 min at 4 °C, then centrifuged at 1,000 xg for 5 min, cells then were re-suspended with 500 µL of 5 mM MgCl_2_ in PBS. 2): non-transfected HEK293, MCF7 and MDA-MB-415 cells were incubated with anti-MGB2 and MGB1 primary monoclonal antibodies (1:400 of 100 µg, anti-MGB2 with MCF7, anti MGB1 with MDA-MB-415, and each of them with HEK293) for 30 min at RT. Cells then were centrifuged 1,000 xg for 5 min and washed three times with PBS. Goat anti mouse FITC labelled secondary antibody (1:500 of 100 µg) was added for 20 min at RT. Finally, cells were centrifuged and washed as above, then re-suspended with 500 µL of PBS. 3): transfected HEK293 were incubated with either 300 nM 6-FAM labelled aptamers, or unlabeled aptamers, or random sequence for 30 min at 4 °C, then they were incubated with anti-MGB2 or MGB1 primary monoclonal antibodies, then goat anti-mouse FITC labelled secondary antibody, applying same conditions as in 2). All cells were studied using flow cytometry by counting 10,000 events and data were analyzed using the flow cytometry C6 sampler software. The fluorescence intensity of goat anti mouse secondary antibody alone was subtracted from the samples where primary and secondary antibodies were used together to obtain the fluorescence intensity for the primary antinodes alone.

### Statistical analysis

All data represent the average ± S.E.M. The binding affinity (K_d_) graphed were plotted using GraphPad Prism software (USA). The statistical significance was determined using a one-way ANOVA followed by the Fisher LSD test using SPSS software (SPSS, version 23). A p value of less than 0.05 was considered to be significant. All plasma and whole blood lysate experiments results were plotted using MS-Excel 2013. BD Accuri™ C6 Software (Version 227) was used to analyze all flow cytometry data.

## Electronic supplementary material

Supplementary Information
