# Supplementary material for: In vitro selections of mammaglobin A and mammaglobin B aptamers for the recognition of circulating breast tumor cells
Source: Sci Rep. 2017 Nov 3;7:14487. doi: 10.1038/s41598-017-13751-z (PMC5670216; doi:10.1038/s41598-017-13751-z)
Supplement: Supplementary file 1 — Supplementary Information [file 41598_2017_13751_MOESM1_ESM.pdf]

In vitro selections of mammaglobin A and mammaglobin B aptamers for the recognition of circulating breast tumor cells

Eman M. Hassan [a, d], William G. Willmore [b,c], Bruce C. McKay [b,c], and Maria C. DeRosa *[b, d]

[a] Institut national de la recherche scientifique - Energie, Materiaux Telecommunication

1650 boul. Lionel-Boulet, Varennes, Quebec, Canada J3X 1S2

[b] Institute of Biochemistry, Carleton University, 1125 Colonel By Drive, Ottawa, Ontario, Canada K1S 5B6

[c] Department of Biology, Carleton University, 1125 Colonel By Drive, Ottawa, Ontario, Canada K1S 5B6

[d] Department of Chemistry, Carleton University, 1125 Colonel By Drive, Ottawa, Ontario, Canada K1S 5B6

*maria.derosa@carleton.ca

Supplementary data

Investigation the expression of MGB2 and MGB1 using ELISA kit

The expression of MGB2 and MGB1 proteins was tested in cell lysate of cell-SELEX cell lines using the commercially available MGB2 and MGB1 ELISA kits. Briefly 100 µL (20 µg) of each lysate was loaded onto the ELISA plate wells (pre-coated with streptavidin monoclonal antibody against MGB2 and MGB1) and incubated for 2 hrs at 37˚C. A biotinylated secondary antibody or HRP-avidin was added for 1 hr at 37˚C and Tetramethylbenzidine (TMB) was used as the substrate. Color development was assessed using microplate reader (BioTek, Canada).

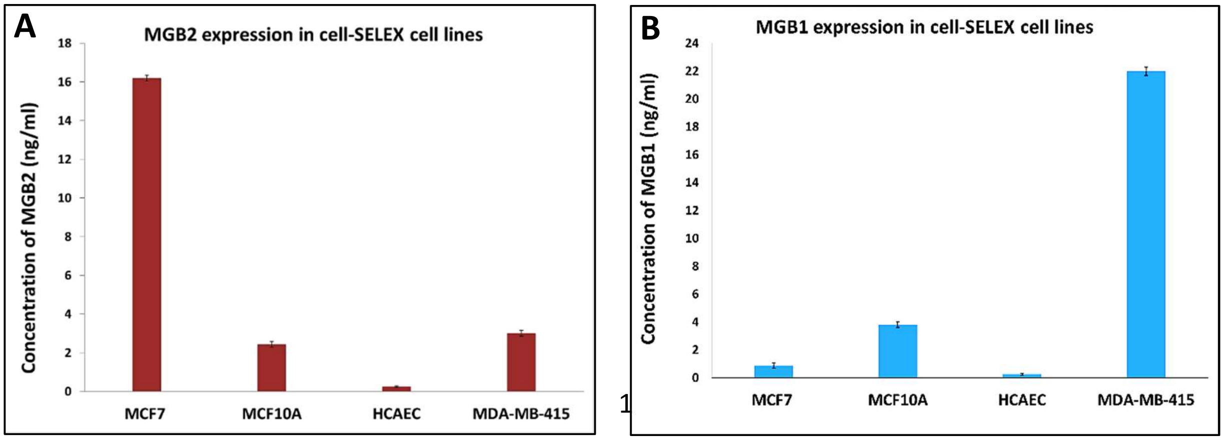

Figure S1. **MGB2 and MGB1 expression of the lysate of all cell lines used in cell-SELEX**. Highest expression of MGB2 and MGB1 proteins was observed in MCF7 (A) and MDA-MB-415

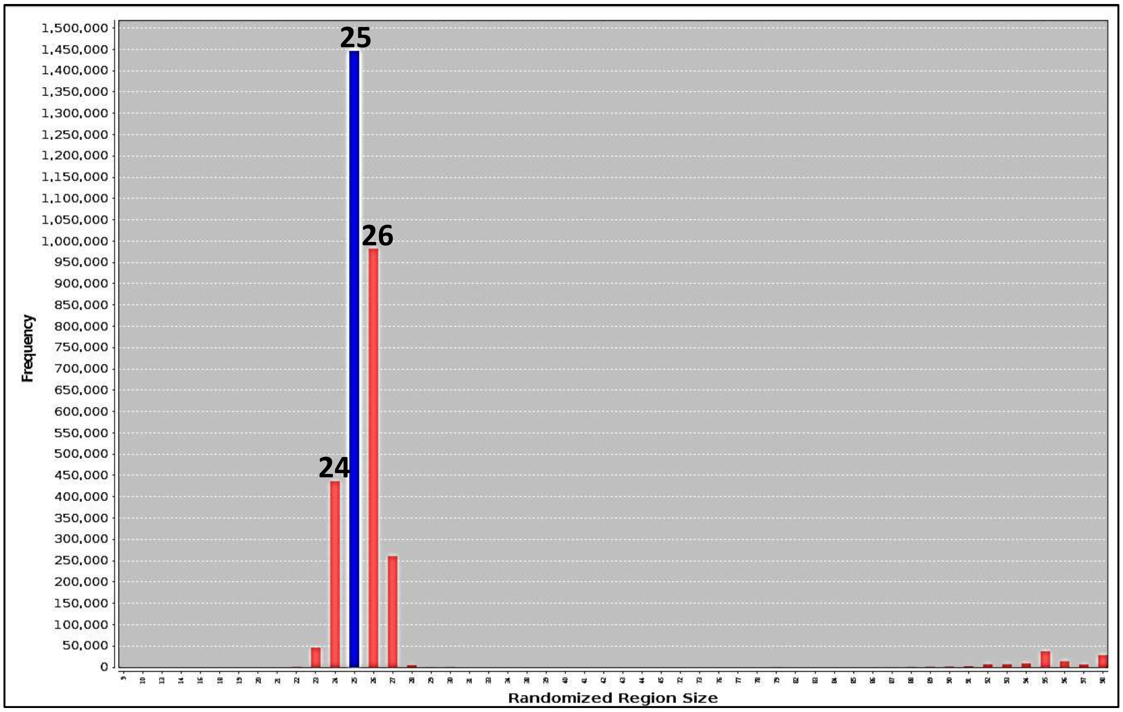

Figure S2. Distribution analysis of the random region size using AptaCluster. (A) Last round of MCF7 (MGB2) showed the dominant size of random region (Blue) is 25 bases and other sizes (red) 24 and 26.

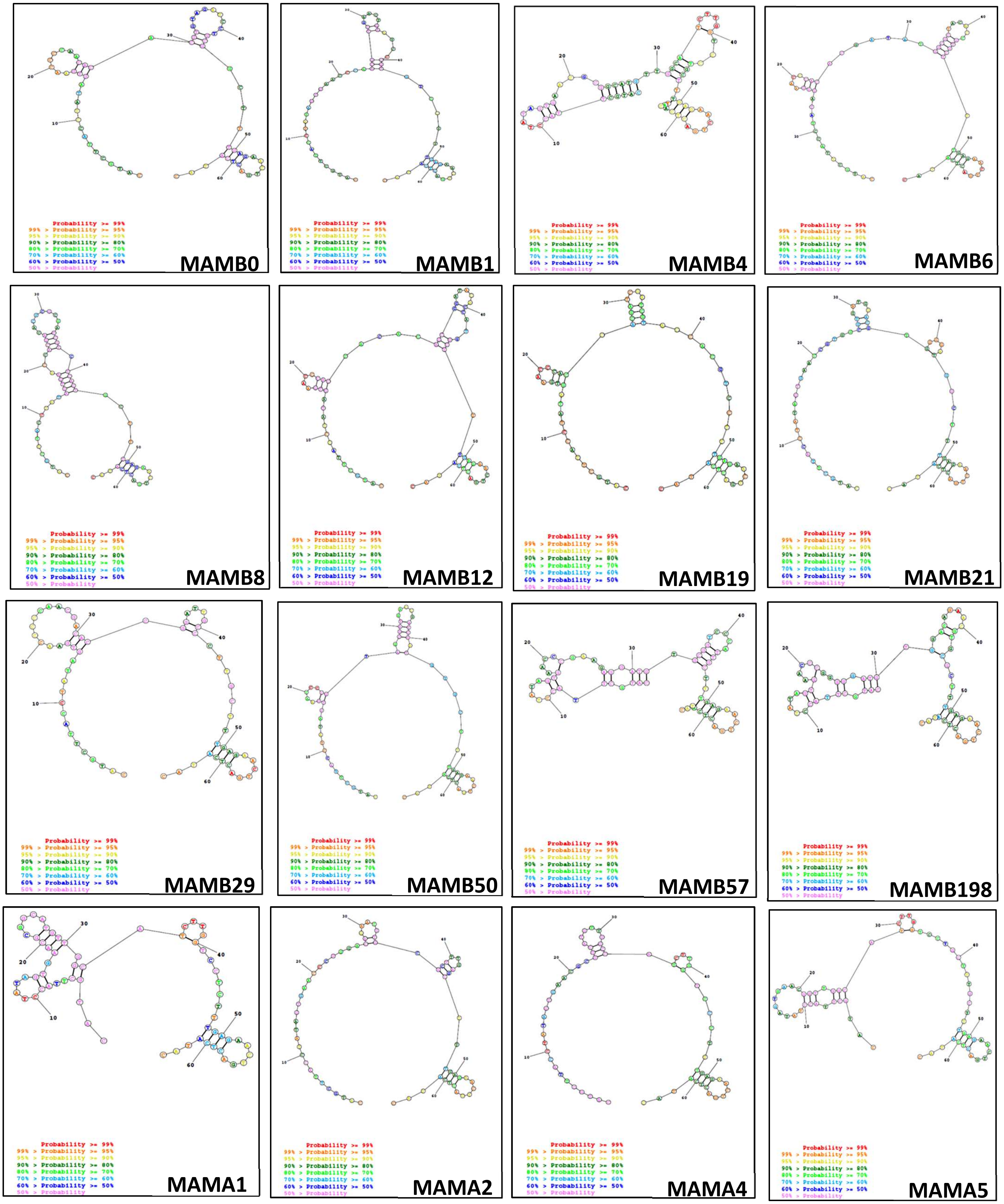
Probability >= 99%
99% > Probability >= 95%
95% > Probability >= 90%
90% > Probability >= 80%
80% > Probability >= 70%
70% > Probability >= 60%
60% > Probability >= 50%
50% > Probability
MAMB0
MAMB1
MAMB4
MAMB6
MAMB8
MAMB12
MAMB19
MAMB21
MAMB29
MAMB50
MAMB57
MAMB198
MAMA1
MAMA2
MAMA4
MAMA5

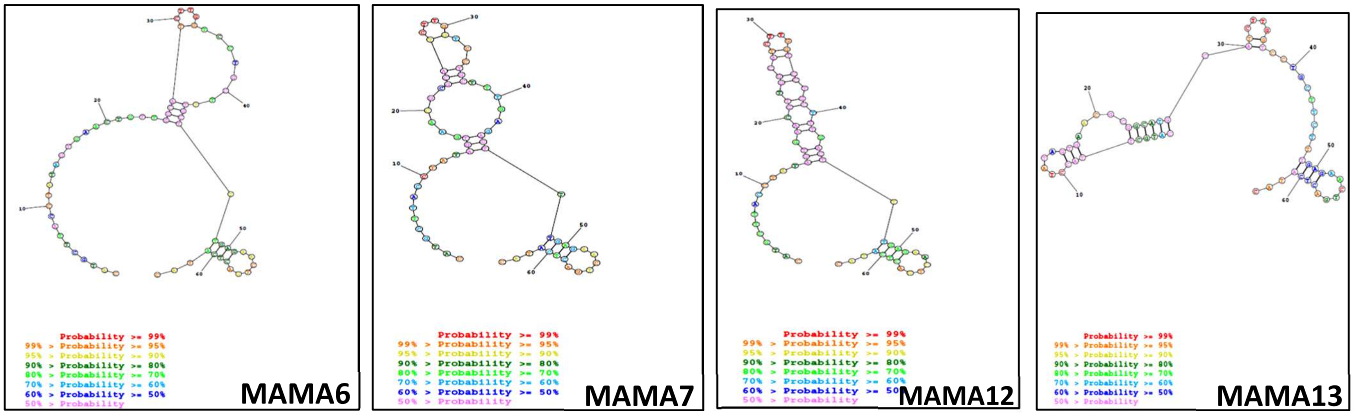

Figure S3. The predicated secondary structure of the chosen aptamers of MGB2 and MB1 using RNAstructure software.

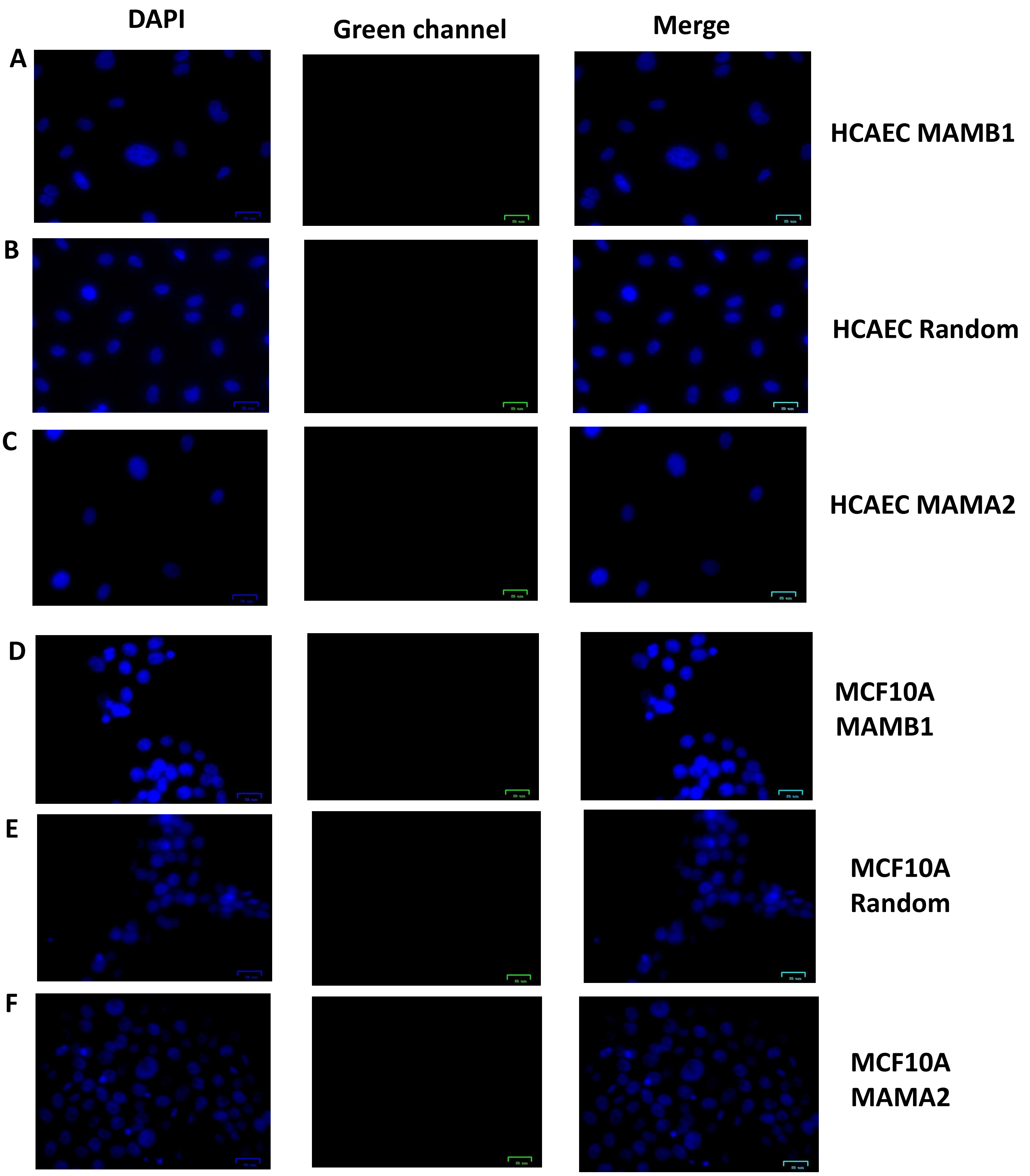
DAPI
Green channel
Merge
A
B
C
D
E
F
HCAEC MAMB1
HCAEC Random
HCAEC MAMA2
MCF10A
MAMB1
MCF10A
Random
MCF10A
MAMA2

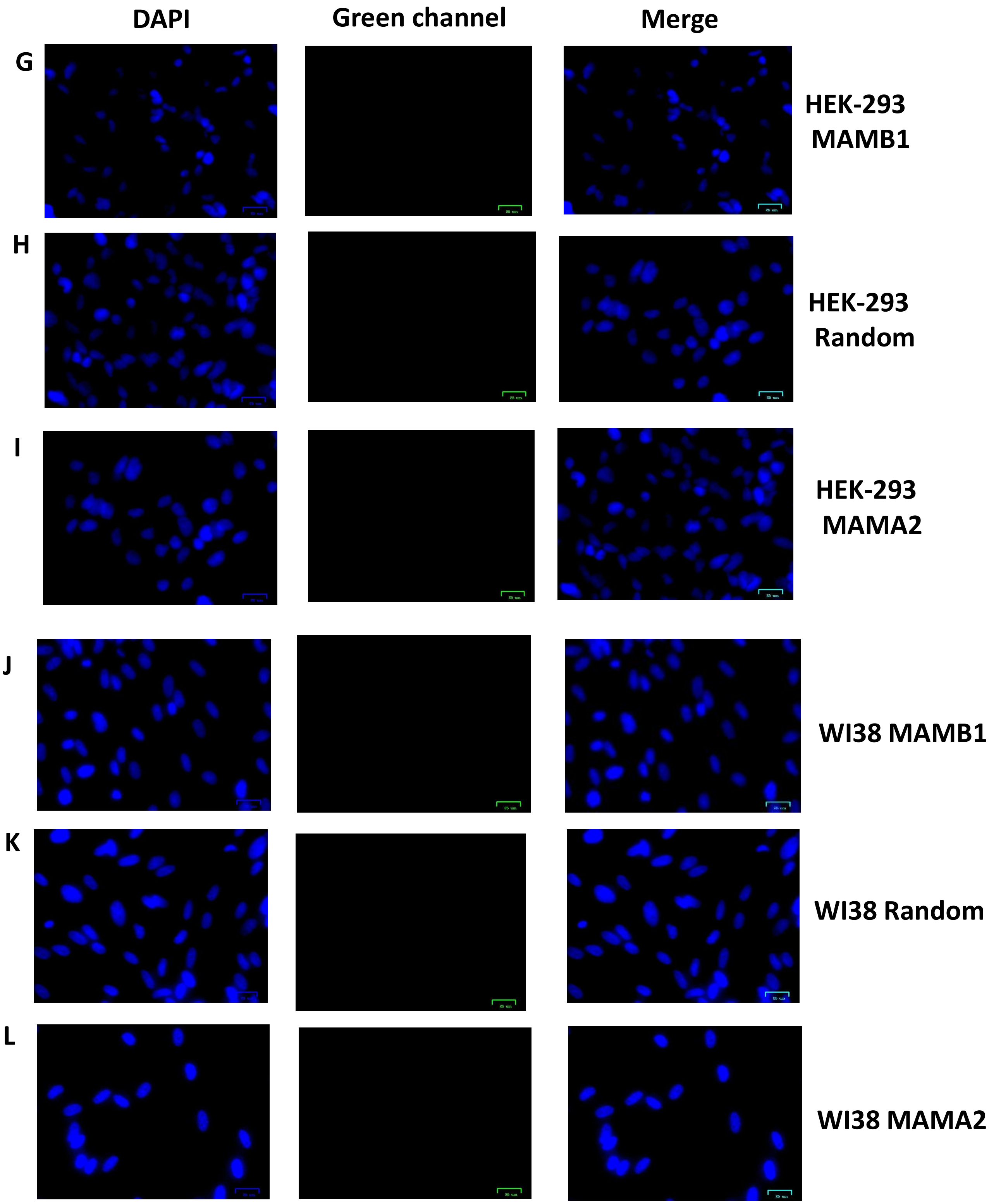
DAPI
Green channel
Merge
G
HEK-293 MAMB1
H
HEK-293 Random
I
HEK-293 MAMA2
J
WI38 MAMB1
K
WI38 Random
L
WI38 MAMA2

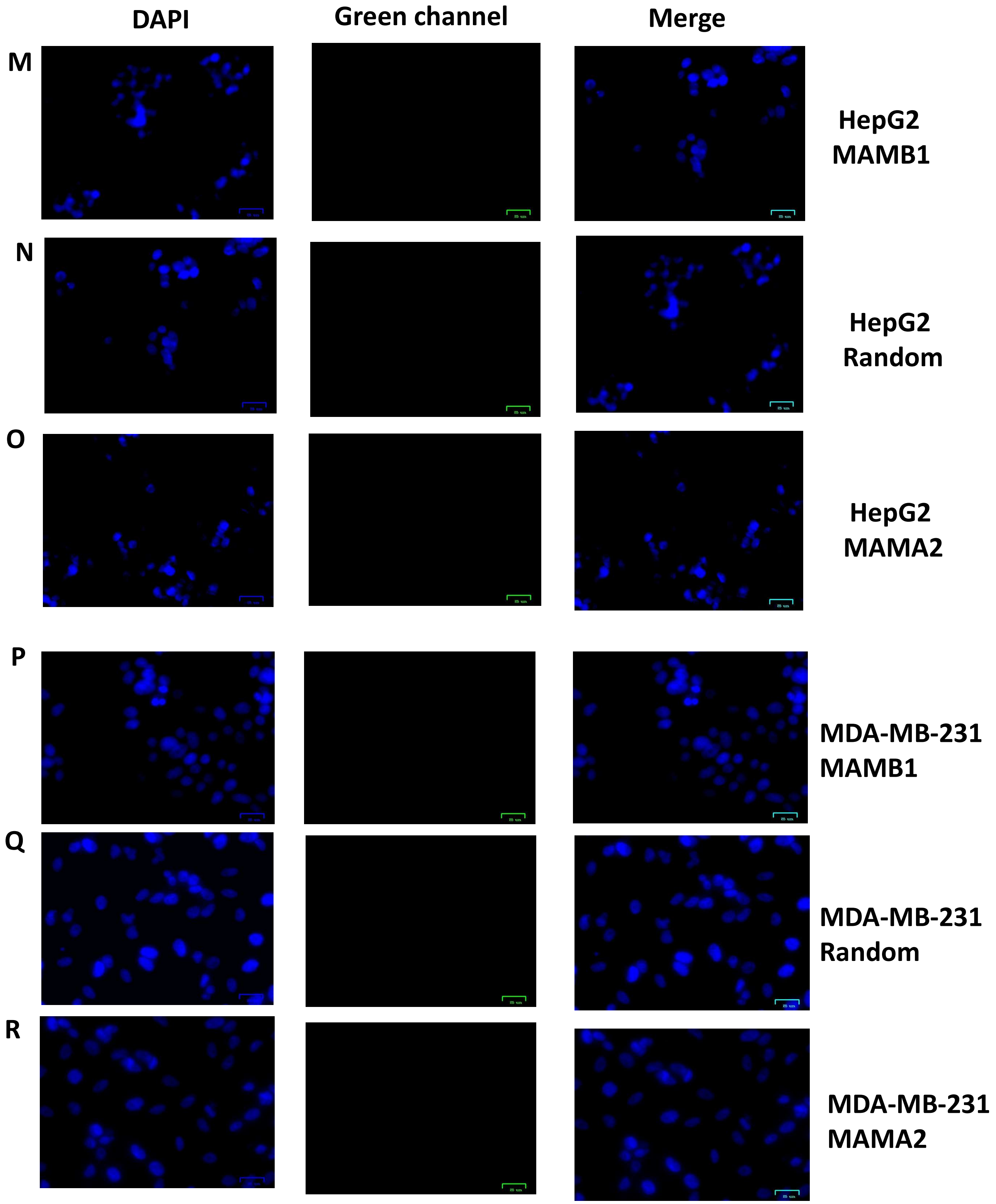
DAPI
Green channel
Merge
M
HepG2
MAMB1
N
HepG2
Random
O
HepG2
MAMA2
P
MDA-MB-231
MAMB1
Q
MDA-MB-231
Random
R
MDA-MB-231
MAMA2

Figure S4. Fluorescence microscopy images of aptamers MAMB1 and MAMA2 binding to counter cancer cell lines used in SELEX and other cancer and normal cell lines. (A-C) MAMB1 binding to HCAEC cells, random sequence, and MAMA2 respectively. **(D-F)** MAMB1 binding to MCF10A cells, random sequence, and MAMA2 respectively. **(G-I)** MAMB1 binding to HEK293 cells, random sequence, and MAMA2 respectively. **(J-L)** MAMB1 binding to WI38 cells, random sequence, and MAMA2 respectively. **(M-O)** MAMB1 binding to HepG2 cells, random sequence, and MAMA2 respectively. **(P-R)** MAMB1 binding to MDA-MB-231 cells, random sequence, and MAMA2 respectively. Both aptamers and the random sequence were incubated with the cells for 30 min and then imaged. Scale bars correspond to 25μm in all images.

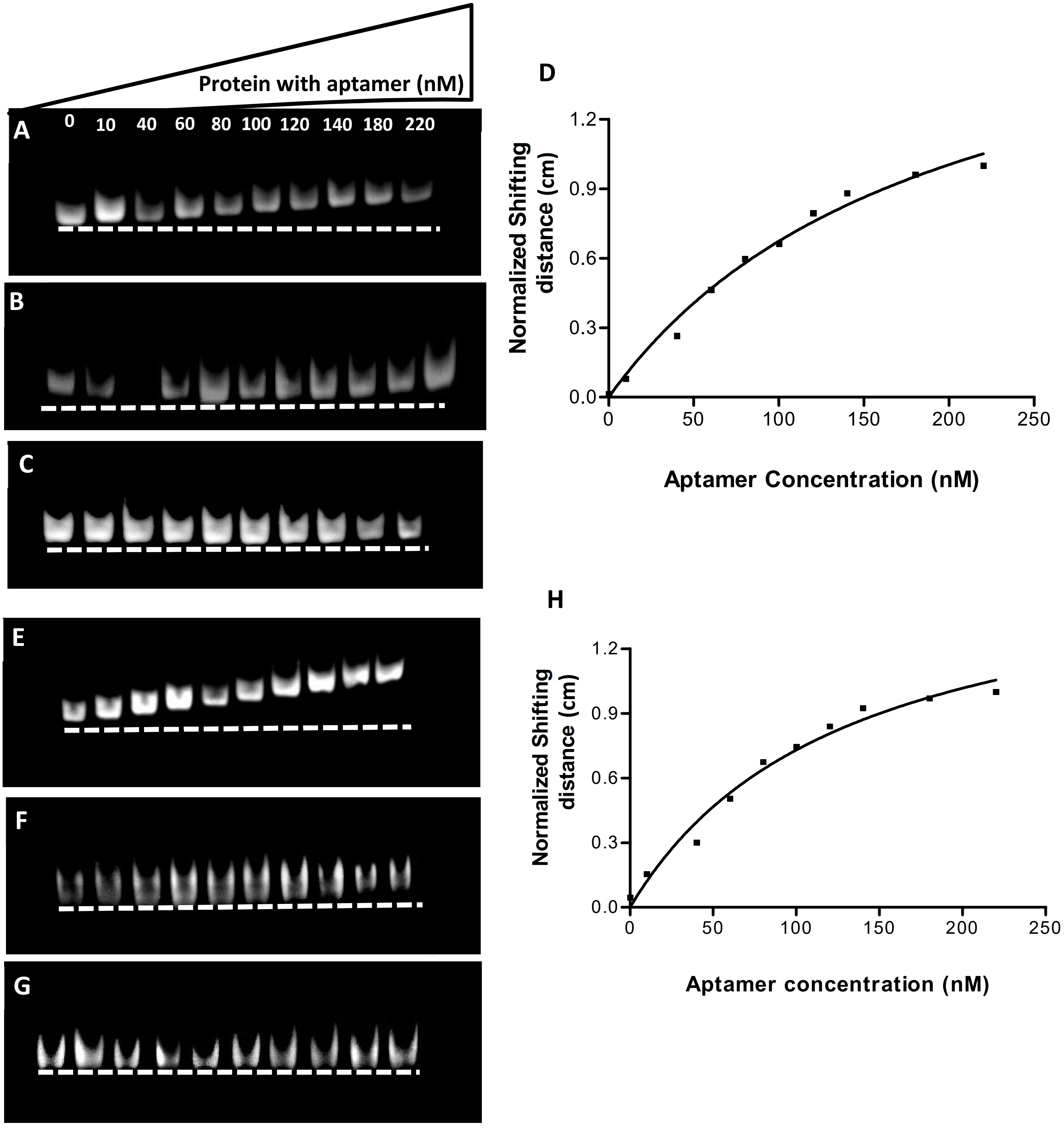

Figure S5. EMSA gel after electrophoresis: (A) EMSA for MGB2 protein with MAMB1 aptamer. (B) EMSA for MGB2 protein with DNA control aptamer. (C) EMSA for BSA protein with MAMB1 (D) plotting curve of (A) MAMB1 (0-220nM) against the shifting distance of MAMB1-MGB2 complex compared to the control (0nM). The Kd value is 95±7.0 as obtained from GraphPad Prism software. (E) EMSA for MGB1 protein with MAMA2 aptamer. (F) EMSA for MGB1 protein with DNA control aptamer. (G) EMSA for BSA protein with MAMA2 (H) plotting curve of (E) MAMA2 (0-220nM) against the shifting distance of MAMA2-MGB1 complex compared to the control (0nM) The Kd value is 88±6.0 as obtained from GraphPad Prism software. Aptamers (600nM) and different concentrations of proteins were incubated at 37C for one hour then EMSA was applied using 8% native PAGE. The distance of the complex compared to the control (0nM) was measured using the analysis tool in the Alpha-imager software. The brightness of gel images was improved by Image J software.

Table S1: Aptamer sequences of MGB2 and MGB1 aptamers where K_d value were not determined. Primer sites are underlined.

Aptamer	Aptamer sequence (5'-3')
MAMB6	CATGCTTACCTATAGTGAACCCACACGATATGGCGCTACACCGTGCTTTGAGAACTGACTCATAC
MAMB19	CATGCTTACCTATAGTGAACCCACCACGATCGATCGTACCACGTCCTTTGAGAACTGACTCATAC
MAMB21	CATGCTTACCTATAGTGAACCGTGGCGTGATGGACGTGGGGATGGCTTTGAGAACTGACTCATAC
MAMB29	CATGCTTACCTATAGTGAACCCCCAACGACACCGGATTGCCCTTGCTTTGAGAACTGACTCATAC
MAMB50	CATGCTTACCTATAGTGAACCCCGTAGCGATGACCGATCGATGTGCTTTGAGAACTGACTCATAC
MAMB198	CATGCTTACCTATAGTGAACCCGGGGGATGTGGACAGAACGTGCGCTTTGAGAACTGACTCATAC
MAMA1	CATGCTTACCTATAGTGAACGCGGCATGTTGGCATCTTGGTCCTCTTTGAGAACTGACTCATAC
MAMA4	CATGCTTACCTATAGTGAACGCGGCATGTTGGCCATCTTGGTCCCTTTGAGAACTGACTCATAC
MAMA7	CATGCTTACCTATAGTGAACGCGCATCTTGGTCCTGCTTTGAGACTTTGAGAACTGACTCATAC
MAMA13	CATGCTTACCTATAGTGAACGCGGCATGCATCTTGGTCCTGCTTCTTTGAGAACTGACTCATAC

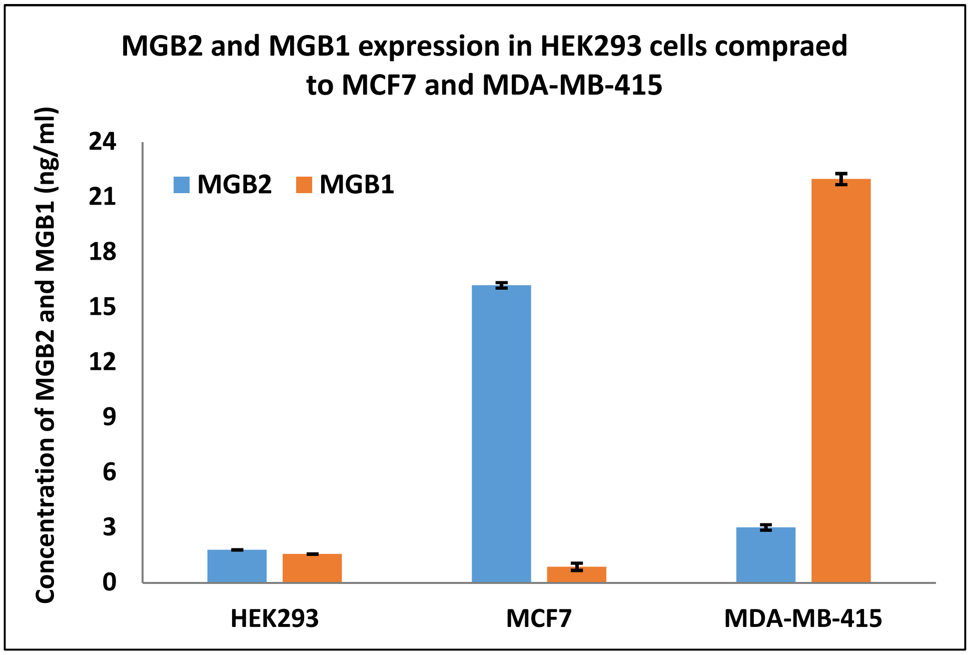

Figure S6: MGB2 and MGB1 expression of HEK293, MCF7, and MDA-MB-415 lysates. HEK293 cells showed low level of MGB2 and MGB1 (1.78 and 1.56 ng/mL respectively) compared to MCF7 (16.19 and 0.87ng/ml for MGB2 and MGB1 respectively) and MDA-MB-415 (3.01 and 21.98ng/ml for MGB2 and MGB1 respectively). The expression of MGB2 and MGB1 was measured using an ELISA kits for both proteins as in Fig. S1.

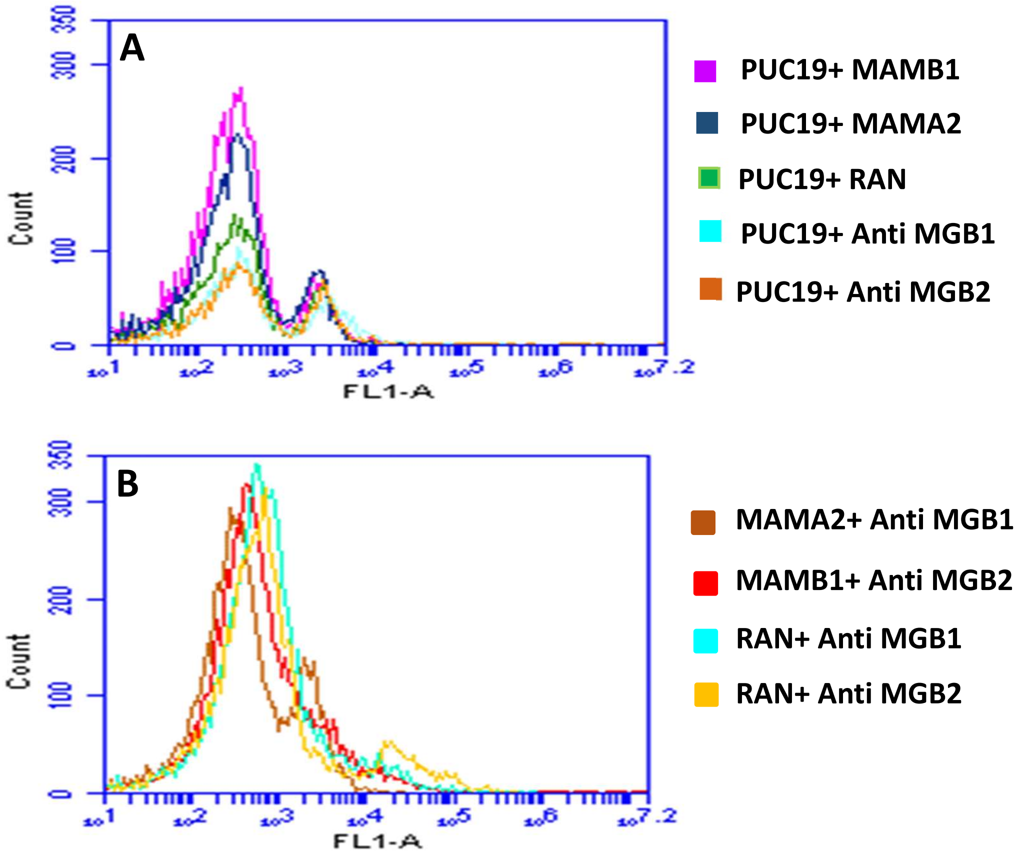

Figure S7: Competition binding assays of MAMB1, MAMA2 aptamers and anti MGB, anti MGB1 antibodies on transfected HEK293 cells with PUC19 empty plasmid. **(A)** Mean of fluorescence intensity of transfected HEK293 with PUC19 plasmid binding to MAMB1 (Pink), MAMA2 (blue), random sequence (green), anti MGB2 (orange), and anti MGB1 (light blue). **(B)** Mean of fluorescence intensity of transfected HEK293 with PUC19 plasmid and blocked with unlabeled MAMB1 and MAMA2 aptamers, then probed with anti MGB2 (red), and anti MGB1 (brown) respectively. Yellow Light blue and histograms showed transfected HEK293 with PUC19 blocked with random sequence and then probed with anti MGB2 and anti MGB1 respectively. Cells were incubated with their corresponding probes and 10000 events was counted by flow cytometry. Then data was analyzed using flow cytometry C6 sampler software.
